# Supplementary material for: Estimating the impact of alternative programmatic cotrimoxazole strategies on mortality among children born to mothers with HIV: A modelling study
Source: PLoS Med. 2024 Feb 20;21(2):e1004334. doi: 10.1371/journal.pmed.1004334 (PMC10914273; doi:10.1371/journal.pmed.1004334)

**Supplement**

**Estimating the Impact of Alternative Programmatic Cotrimoxazole Strategies on Mortality Among Children Born to Mothers with HIV: A Modelling Study**

Contents

[Section 1: Model Assumptions and Data Sources 3](#_Toc156480382)

[**Table A. Model Assumptions and Data Sources** 3](#_Toc156480383)

[Section 2: Mortality under alternative cotrimoxazole strategies 8](#_Toc156480384)

[**Fig A. Percentage Mortality Under Alternative Cotrimoxazole Strategies.** Predicted mortality percentage per year by country under alternative cotrimoxazole strategies. 8](#_Toc156480385)

[**Fig B. Excess Mortality Rate Under Alternative Cotrimoxazole Strategies.** Predicted excess mortality rate (per 100 000) per year by country under alternative cotrimoxazole strategies. 9](#_Toc156480386)

[**Fig C. Risk Ratios Under Alternative Cotrimoxazole Strategies.** Predicted risk ratio per year by country under alternative cotrimoxazole strategies. 9](#_Toc156480387)

[Section 3: Combined Sensitivity Analysis 10](#_Toc156480388)

[**Fig D. Sensitivity Analysis for Zimbabwe (Risk Ratios).** Sensitivity analysis, for Zimbabwe, exploring the effect of varying assumptions on the Risk Ratio for deaths (6 weeks to 2 years) compared to the current WHO strategy 10](#_Toc156480389)

[**Fig E. Sensitivity Analysis for Zimbabwe (Excess Deaths).** Sensitivity analysis, for Zimbabwe, exploring the effect of varying assumptions on the Risk Ratio for deaths (6 weeks to 2 years) compared to the current WHO strategy 10](#_Toc156480390)

[**Fig F. Sensitivity Analysis for Cote d’Ivoire (Risk Ratios).** Sensitivity analysis, for Cote d’Ivoire, exploring the effect of varying assumptions on the Risk Ratio for deaths (6 weeks to 2 years) compared to the current WHO strategy 11](#_Toc156480391)

[**Fig G. Sensitivity Analysis for Cote d’Ivoire (Excess Deaths).** Sensitivity analysis, for Cote d’Ivoire, exploring the effect of varying assumptions on the Risk Ratio for deaths (6 weeks to 2 years) compared to the current WHO strategy 11](#_Toc156480392)

[**Fig H. Sensitivity Analysis for Mozambique (Risk Ratios).** Sensitivity analysis, for Mozambique, exploring the effect of varying assumptions on the Risk Ratio for deaths (6 weeks to 2 years) compared to the current WHO strategy 12](#_Toc156480393)

[**Fig I. Sensitivity Analysis for Uganda (Risk Ratios).** Sensitivity analysis, for Uganda, exploring the effect of varying assumptions on the Risk Ratio for deaths (6 weeks to 2 years) compared to the current WHO strategy 13](#_Toc156480394)

[**Fig J. Sensitivity Analysis for Uganda (Excess Deaths).** Sensitivity analysis, for Uganda, exploring the effect of varying assumptions on the Risk Ratio for deaths (6 weeks to 2 years) compared to the current WHO strategy 13](#_Toc156480395)

[Section 4: Sensitivity Analysis for Key Assumptions (Individual Variables) 14](#_Toc156480396)

[**Fig K. Sensitivity Variable - Cotrimoxazole Uptake, Zimbabwe.** Risk ratio of mortality for varying risk reduction from cotrimoxazole uptake for Zimbabwe, (Risk Ratio). 14](#_Toc156480397)

[**Fig L. Sensitivity Variable – Risk Reduction from CTX, Zimbabwe.** Risk ratio of mortality for varying risk reduction from cotrimoxazole while infant with HIV is taking antiretroviral therapy for Zimbabwe, (Risk Ratio). Risk reduction from 15%-43%. 14](#_Toc156480398)

[**Fig M. Sensitivity Variable – Risk Reduction from CTX, Zimbabwe.** Risk ratio of mortality for varying risk reduction from cotrimoxazole while infant with HIV is taking antiretroviral therapy for Zimbabwe, (Risk Ratio). Risk Reduction from 25%-60%. 15](#_Toc156480399)

[**Fig N. Sensitivity Variable – EID Testing, Zimbabwe.** Risk ratio of mortality for varying probability of HIV-exposed infants undergoing Early Infant Diagnosis (EID) for Zimbabwe, (Risk Ratio). 15](#_Toc156480400)

[**Fig O. Sensitivity Variable – Perinatal MTCT, Zimbabwe.** Risk ratio of mortality for varying probability of perinatal mother-to-child transmission (MTCT) for Zimbabwe, (Risk Ratio). 16](#_Toc156480401)

[**Fig P. Sensitivity Variable – Postnatal MTCT, Zimbabwe.** Risk ratio of mortality for varying probability of post-natal mother-to-child transmission (MTCT) for Zimbabwe, (Risk Ratio). 16](#_Toc156480402)

[**Fig Q. Sensitivity Variable - Cotrimoxazole Uptake, Cote d’Ivoire.** Risk ratio of mortality for varying risk reduction from cotrimoxazole uptake for Cote d’Ivoire, (Risk Ratio). 17](#_Toc156480403)

[**Fig R. Sensitivity Variable – Risk Reduction from CTX, Cote d’Ivoire.** Risk ratio of mortality for varying risk reduction from cotrimoxazole while infant with HIV is taking antiretroviral therapy for Cote d’Ivoire, (Risk Ratio). Risk reduction from 15%-43%. 17](#_Toc156480404)

[**Fig S. Sensitivity Variable – Risk Reduction from CTX, Cote d’Ivoire.** Risk ratio of mortality for varying risk reduction from cotrimoxazole while infant with HIV is taking antiretroviral therapy for Cote d’Ivoire, (Risk Ratio). Risk reduction from 15%-60%. 18](#_Toc156480405)

[**Fig T. Sensitivity Variable – EID Testing, Cote d’Ivoire.** Risk ratio of mortality for varying probability of HIV-exposed infants undergoing Early Infant Diagnosis (EID) for Cote d’Ivoire, (Risk Ratio). 18](#_Toc156480406)

[**Fig U. Sensitivity Variable – Perinatal MTCT, Cote d’Ivoire.** Risk ratio of mortality for varying probability of perinatal mother-to-child transmission (MTCT) for Cote d’Ivoire, (Risk Ratio). 19](#_Toc156480407)

[**Fig V. Sensitivity Variable – Postnatal MTCT, Cote d’Ivoire.** Risk ratio of mortality for varying probability of post-natal mother-to-child transmission (MTCT) for Cote d’Ivoire, (Risk Ratio). 19](#_Toc156480408)

[**Fig W. Sensitivity Variable - Cotrimoxazole Uptake, Mozambique.** Risk ratio of mortality for varying risk reduction from cotrimoxazole uptake for Mozambique, (Risk Ratio). 20](#_Toc156480409)

[**Fig X. Sensitivity Variable – Risk Reduction from CTX, Mozambique.** Risk ratio of mortality for varying risk reduction from cotrimoxazole while infant with HIV is taking antiretroviral therapy for Mozambique, (Risk Ratio). Risk reduction from 15%-43%. 20](#_Toc156480410)

[**Fig Y. Sensitivity Variable – Risk Reduction from CTX, Mozambique.** Risk ratio of mortality for varying risk reduction from cotrimoxazole while infant with HIV is taking antiretroviral therapy for Mozambique, (Risk Ratio). Risk reduction from 15%-60%. 21](#_Toc156480411)

[**Fig Z. Sensitivity Variable – EID Testing, Mozambique.** Risk ratio of mortality for varying probability of HIV-exposed infants undergoing Early Infant Diagnosis (EID) for Mozambique, (Risk Ratio). 21](#_Toc156480412)

[**Fig AA. Sensitivity Variable – Perinatal MTCT, Mozambique.** Risk ratio of mortality for varying probability of perinatal mother-to-child transmission (MTCT) for Mozambique, (Risk Ratio). 22](#_Toc156480413)

[**Fig AB. Sensitivity Variable – Postnatal MTCT, Mozambique.** Risk ratio of mortality for varying probability of post-natal mother-to-child transmission (MTCT) for Mozambique, (Risk Ratio). 22](#_Toc156480414)

[**Fig AC. Sensitivity Variable - Cotrimoxazole Uptake, Uganda.** Risk ratio of mortality for varying risk reduction from cotrimoxazole uptake for Uganda, (Risk Ratio). 23](#_Toc156480415)

[**Fig AD. Sensitivity Variable – Risk Reduction from CTX, Uganda.** Risk ratio of mortality for varying risk reduction from cotrimoxazole while infant with HIV is taking antiretroviral therapy for Uganda, (Risk Ratio). Risk reduction from 15%-43% 23](#_Toc156480416)

[**Fig AE. Sensitivity Variable – Risk Reduction from CTX, Uganda.** Risk ratio of mortality for varying risk reduction from cotrimoxazole while infant with HIV is taking antiretroviral therapy for Uganda, (Risk Ratio). Risk reduction from 15%-60%. 24](#_Toc156480417)

[**Fig AF. Sensitivity Variable – EID Testing, Uganda.** Risk ratio of mortality for varying probability of HIV-exposed infants undergoing Early Infant Diagnosis (EID) for Uganda, (Risk Ratio). 24](#_Toc156480418)

[**Fig AG. Sensitivity Variable – Perinatal MTCT, Uganda.** Risk ratio of mortality for varying probability of perinatal mother-to-child transmission (MTCT) for Uganda, (Risk Ratio). 25](#_Toc156480419)

[**Fig AH. Sensitivity Variable – Postnatal MTCT, Uganda.** Risk ratio of mortality for varying probability of post-natal mother-to-child transmission (MTCT) for Uganda, (Risk Ratio). 25](#_Toc156480420)

# Section 1: Model Assumptions and Data Sources

## **Table A.** **Model Assumptions and Data Sources**

| **Type** | **Assumption** | **Value** | **Source** | **Range of values for sensitivity analysis** | **Comments and Caveats** |
| --- | --- | --- | --- | --- | --- |
| HIV status | Probability child born with HIV (by 6 weeks) – Perinatal MTCT | ZWE 5%  CIV 4%  MOZ 6%  UGA 3% | 2021 Start Free, Stay Free | ZWE 4%, 3%, 2%, 1%, 0%  CIV 3%, 2%, 1%, 0%  MOZ 5%, 4%, 3%, 2%, 1%, 0%  UGA 3%, 2%, 1%, 0% | Data may not reflect current vertical transmission rates. Lower transmission rates interrogated in sensitivity analysis |
| HIV status | Cumulative probability child acquires HIV postnatally by 9-month test if breastfed – Postnatal MTCT | ZWE 1.6%  CIV 1.7%  MOZ 3.4%  UGA 1.3% | Weekly transmission rates derived from UNAIDS Reference Data 2021 | ZWE 1.1%, 0.6%, 0.1%, 0%  CIV 1.2%, 0.7%, 0.2%, 0%  MOZ 2.9%, 2.4%, 1.9%, 1.4%, 0.9%, 0.4%, 0%  UGA 0.8% | Calculated assuming weekly transmission rates (between 6 weeks and 9 months) are constant. |
| HIV status | Cumulative probability child acquires HIV postnatally if breastfed by end of breastfeeding – Postnatal MTCT | ZWE 2.0%  CIV 2.1%  MOZ 4.1%  UGA 1.6% | Weekly transmission rates derived from UNAIDS Reference Data 2021 | ZWE 2.0%, 1.5%, 1.0%, 0.5%, 0%  CIV 2.1%, 1.6%, 1.1%, 0.6%, 0.1%, 0%  MOZ 4.1%, 3.6%, 3.1%, 2.6%, 2.1%, 1.6%, 1.1%, 0.6%, 0.1%, 0%  UGA 1.6%, 1.1%, 0.6%, 0.1%, 0% | Calculated assuming weekly transmission rates (between 6 weeks and 18 months) are constant. |
| HIV status | Cumulative probability child acquires HIV postnatally if breastfed between 9-month test and end of breastfeeding – Postnatal MTCT | ZWE 0.3%  CIV 0.4%  MOZ 0.7%  UGA 0.3% | Calculated from 9 month and end of breastfeeding probabilities |  | Calculated assuming weekly transmission rates (between 9 months and 18 months) are constant. |
| Breastfeeding | Probability breastfed | 100% | Demographic and Health Surveys | Fixed | Based on DHS estimates for available countries (ex. Zimbabwe) where available. |
| Breastfeeding | Length of breastfeeding (months) | 18 months | Demographic and Health Surveys | Fixed | Based on DHS estimates for available countries (ex. Zimbabwe) where available, and current WHO guidelines which assume CTX provision to end of breastfeeding or 18 months. |
| Testing | Probability child has Early HIV test (by 6 weeks) | ZWE 75.9%  CIV 60.8%  MOZ 82.9%  UGA 66.2% | UNAIDS Reference Data 2021 | Increased in 10% increments until 100% | Data may not reflect current EID rates. Higher rates interrogated in sensitivity analysis considering rapid improvement in testing rates. |
| Testing | Probability child has 9-month test if have EID | 80% | Expert Opinion | Fixed | No data available on this conditional probability. Assumed based on expert opinion* as children who have accessed EID testing are more likely to be retained in the test-to-treat cascade than those who have not engaged with EID testing. |
| Testing | Probability child has end of breastfeeding test if have EID | 50% | Expert Opinion | Fixed | No data available on this conditional probability. Assumed based on expert opinion* as children who have accessed EID testing are more likely to be retained in the test-to-treat cascade than those who have not engaged with EID testing. |
| Testing | Time from end of breastfeeding to final test taking place | 1 month | Expert Opinion | Fixed | Data not available on country-specific rates. Expert opinion* which follows WHO guidance on postnatal HIV testing (one month from end of breastfeeding). |
| Testing | Probability child has 9-month test if no EID | 10% | Expert Opinion | Fixed | No data available on this conditional probability. Assumed based on expert opinion* as children who have accessed EID testing are more likely to be retained in the test-to-treat cascade than those who have not engaged with EID testing. |
| Testing | Probability child has end of breastfeeding test if no EID | 30% | Expert Opinion | Fixed | No data available on this conditional probability. Assumed based on expert opinion as children who have accessed EID testing are more likely to be retained in the test-to-treat cascade than those who have not engaged with EID testing. |
| ART use | ART uptake after positive HIV test result | 77% | Luo et al (2022) | Fixed | Data extracted from results of meta-analysis |
| CTX use | Cotrimoxazole uptake | 100% | Expert Opinion* | 40%, 60%, 80% | No publicly available data for settings. Lower uptake rates interrogated. |
| CTX effect | Relative risk for reduction in risk of death by receiving CTX (assumed constant) not on ART | 0.57 | Chintu et al (2004) | 0.85, 0.80, 0.75, 0.70. 0.65, 0.60 | Trial conducted prior to ART availability, in hospitalised children, few infants in study. In sensitivity analysis, a lower mortality benefit was interrogated. |
| Death | Probability child without HIV dies between 6wks-3mths without CTX, given survived to 6wks | ZWE 0.9%  CIV 1.6%  MOZ 0.9%  UGA 0.5% | Arikawa et al (2018) | Fixed | Source study is individual pooled analysis of African and Asian studies for HEU children. Data for given time windows extracted from 24-month survival estimates from birth by geographical region |
| Death | Probability child without HIV dies between 3-6mths without CTX, given survived to 3mths | ZWE 1.2%  CIV 1.4%  MOZ 1.2%  UGA 1.2% | Arikawa et al (2018) | Fixed | Source study is individual pooled analysis of African and Asian studies for HEU children. Data for given time windows extracted from 24-month survival estimates from birth by geographical region |
| Death | Probability child without HIV dies between 6-9mths without CTX, given survived to 6mths | ZWE 1.0%  CIV 0.6%  MOZ 1.0%  UGA 0.7% | Arikawa et al (2018) | Fixed | Source study is individual pooled analysis of African and Asian studies for HEU children. Data for given time windows extracted from 24-month survival estimates from birth by geographical region |
| Death | Probability child without HIV dies between 9-12mths without CTX, given survived to 9mths | ZWE 0.8%  CIV 0.1%  MOZ 0.8%  UGA 0.8% | Arikawa et al (2018) | Fixed | Source study is individual pooled analysis of African and Asian studies for HEU children. Data for given time windows extracted from 24-month survival estimates from birth by geographical region |
| Death | Probability child without HIV dies between 12-15mths without CTX, given survived to 12mths | ZWE 0.6%  CIV 0.1%  MOZ 0.6%  UGA 0.1% | Arikawa et al (2018) | Fixed | Source study is individual pooled analysis of African and Asian studies for HEU children. Data for given time windows extracted from 24-month survival estimates from birth by geographical region |
| Death | Probability child without HIV dies between 15-18mths without CTX, given survived to 15mths | ZWE 0.2%  CIV 0.4%  MOZ 0.2%  UGA 0.1% | Arikawa et al (2018) | Fixed | Source study is individual pooled analysis of African and Asian studies for HEU children. Data for given time windows extracted from 24-month survival estimates from birth by geographical region |
| Death | Probability child without HIV dies between 18-21mths without CTX, given survived to 18mths | ZWE 0.1%  CIV 0.4%  MOZ 0.1%  UGA 0.1% | Arikawa et al (2018) | Fixed | Source study is individual pooled analysis of African and Asian studies for HEU children. Data for given time windows extracted from 24-month survival estimates from birth by geographical region |
| Death | Probability child without HIV dies between 21-24mths without CTX, given survived to 21mths | ZWE 0.2%  CIV 0.4%  MOZ 0.2%  UGA 0.1% | Arikawa et al (2018) | Fixed | Source study is individual pooled analysis of African and Asian studies for HEU children. Data for given time windows extracted from 24-month survival estimates from birth by geographical region |
| Death | Probability child with HIV dies between 6wks-3mths without CTX or ART, given survived to 6wks | 5.2% | Newell et al (2004) | Fixed | Source study is pooled analysis of 7 studies prior to ART and CTX era. |
| Death | Probability child with HIV dies between 3-6mths without CTX or ART, given survived to 3mths | 13.2% | Newell et al (2004) | Fixed | Source study is pooled analysis of 7 studies prior to ART and CTX era. |
| Death | Probability child with HIV dies between 6-9mths without CTX or ART, given survived to 6mths | 8.3% | Newell et al (2004) | Fixed | Source study is pooled analysis of 7 studies prior to ART and CTX era. |
| Death | Probability child with HIV dies between 9-12mths without CTX or ART, given survived to 9mths | 7.5% | Newell et al (2004) | Fixed | Source study is pooled analysis of 7 studies prior to ART and CTX era. |
| Death | Probability child with HIV dies between 12-15mths without CTX or ART, given survived to 12mths | 5.1% | Newell et al (2004) | Fixed | Source study is pooled analysis of 7 studies prior to ART and CTX era. |
| Death | Probability child with HIV dies between 15-18mths without CTX or ART, given survived to 15mths | 5.5% | Newell et al (2004) | Fixed | Source study is pooled analysis of 7 studies prior to ART and CTX era. |
| Death | Probability child with HIV dies between 18-21mths without CTX or ART, given survived to 18mths | 4.7% | Newell et al (2004) | Fixed | Source study is pooled analysis of 7 studies prior to ART and CTX era. |
| Death | Probability child with HIV dies between 21-24mths without CTX or ART, given survived to 21mths | 1.9% | Newell et al (2004) | Fixed | Source study is pooled analysis of 7 prior to ART and CTX era. |
| Death | Probability child with HIV on ART dies between 6wks-3mths without CTX, given survived to 6wks | 1.248% | 76% mortality reduction applied based on Violari et al (2008) to data from Newell et al (2004) | Fixed | Given no trials where infants with HIV are not also given CTX, mortality reduction from CHER trial applied to natural history studies (conducted prior to CTX use). |
| Death | Probability child with HIV on ART dies between 3-6mths without CTX, given survived to 3mths | 3.168% | 76% mortality reduction applied based on Violari et al (2008) to data from Newell et al (2004) | Fixed | Given no trials where infants with HIV are not also given CTX, mortality reduction from CHER trial applied to natural history studies (conducted prior to CTX use). |
| Death | Probability child with HIV on ART dies between 6-9mths without CTX, given survived to 6mths | 1.992% | 76% mortality reduction applied based on Violari et al (2008) to data from Newell et al (2004) | Fixed | Given no trials where infants with HIV are not also given CTX, mortality reduction from CHER trial applied to natural history studies (conducted prior to CTX use). |
| Death | Probability child with HIV on ART dies between 9-12mths without CTX, given survived to 9mths | 1.800% | 76% mortality reduction applied based on Violari et al (2008) to data from Newell et al (2004) | Fixed | Given no trials where infants with HIV are not also given CTX, mortality reduction from CHER trial applied to natural history studies (conducted prior to CTX use). |
| Death | Probability child with HIV on ART dies between 12-15mths without CTX, given survived to 12mths | 1.224% | 76% mortality reduction applied based on Violari et al (2008) to data from Newell et al (2004) | Fixed | Given no trials where infants with HIV are not also given CTX, mortality reduction from CHER trial applied to natural history studies (conducted prior to CTX use). |
| Death | Probability child with HIV on ART dies between 15-18mths without CTX, given survived to 15mths | 1.320% | 76% mortality reduction applied based on Violari et al (2008) to data from Newell et al (2004) | Fixed | Given no trials where infants with HIV are not also given CTX, mortality reduction from CHER trial applied to natural history studies (conducted prior to CTX use). |
| Death | Probability child with HIV on ART dies between 18-21mths without CTX, given survived to 18mths | 1.128% | 76% mortality reduction applied based on Violari et al (2008) to data from Newell et al (2004) | Fixed | Given no trials where infants with HIV are not also given CTX, mortality reduction from CHER trial applied to natural history studies (conducted prior to CTX use). |
| Death | Probability child with HIV on ART dies between 21-24mths without CTX, given survived to 21mths | 0.456% | 76% mortality reduction applied based on Violari et al (2008) to data from Newell et al (2004) | Fixed | Given no trials where infants with HIV are not also given CTX, mortality reduction from CHER trial applied to natural history studies (conducted prior to CTX use). |

# Section 2: Mortality under alternative cotrimoxazole strategies

## **Fig A. Percentage Mortality Under Alternative Cotrimoxazole Strategies.** Predicted mortality percentage per year by country under alternative cotrimoxazole strategies.


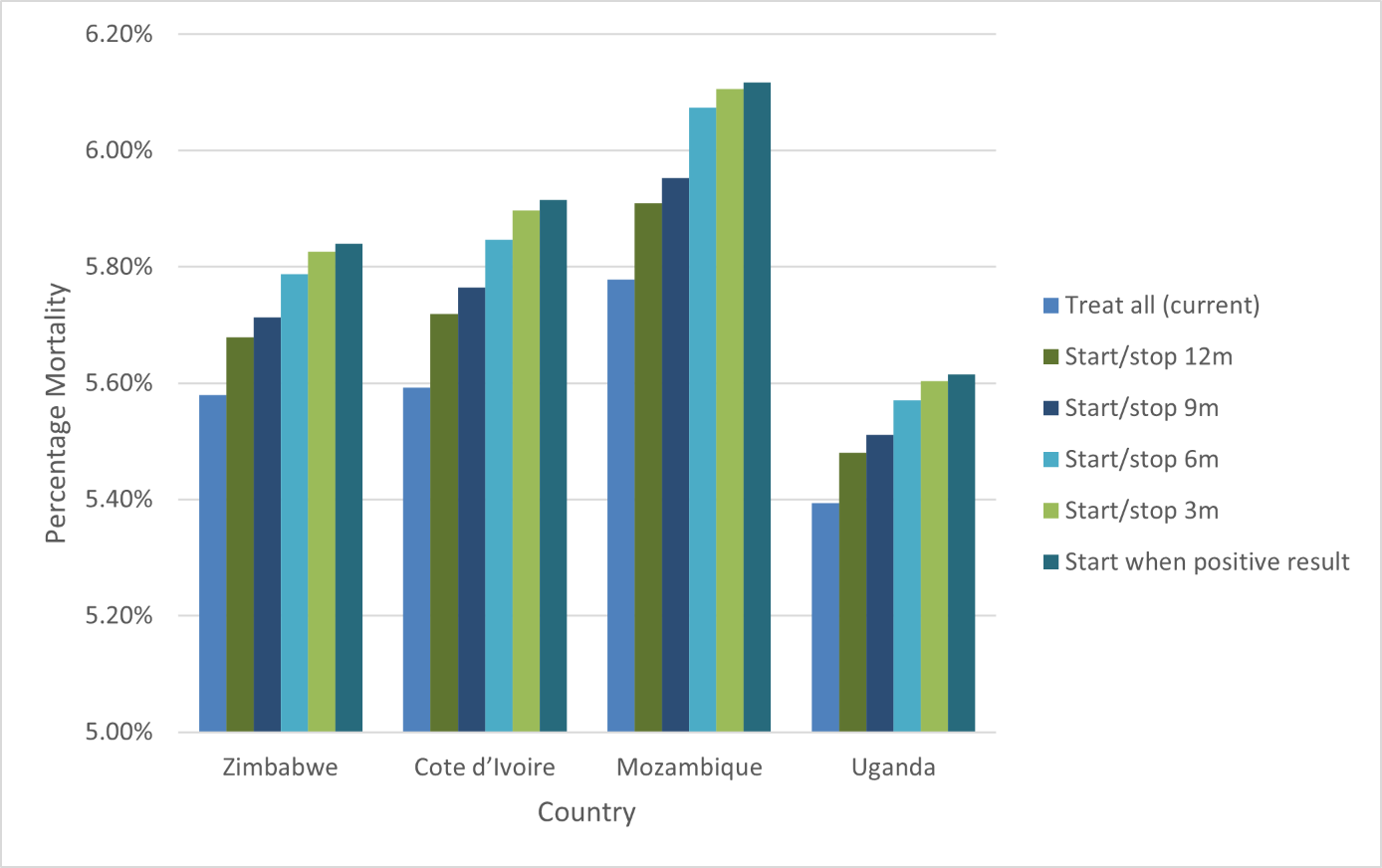


## **Fig B. Excess Mortality Rate Under Alternative Cotrimoxazole Strategies.** Predicted excess mortality rate (per 100 000) per year by country under alternative cotrimoxazole strategies.


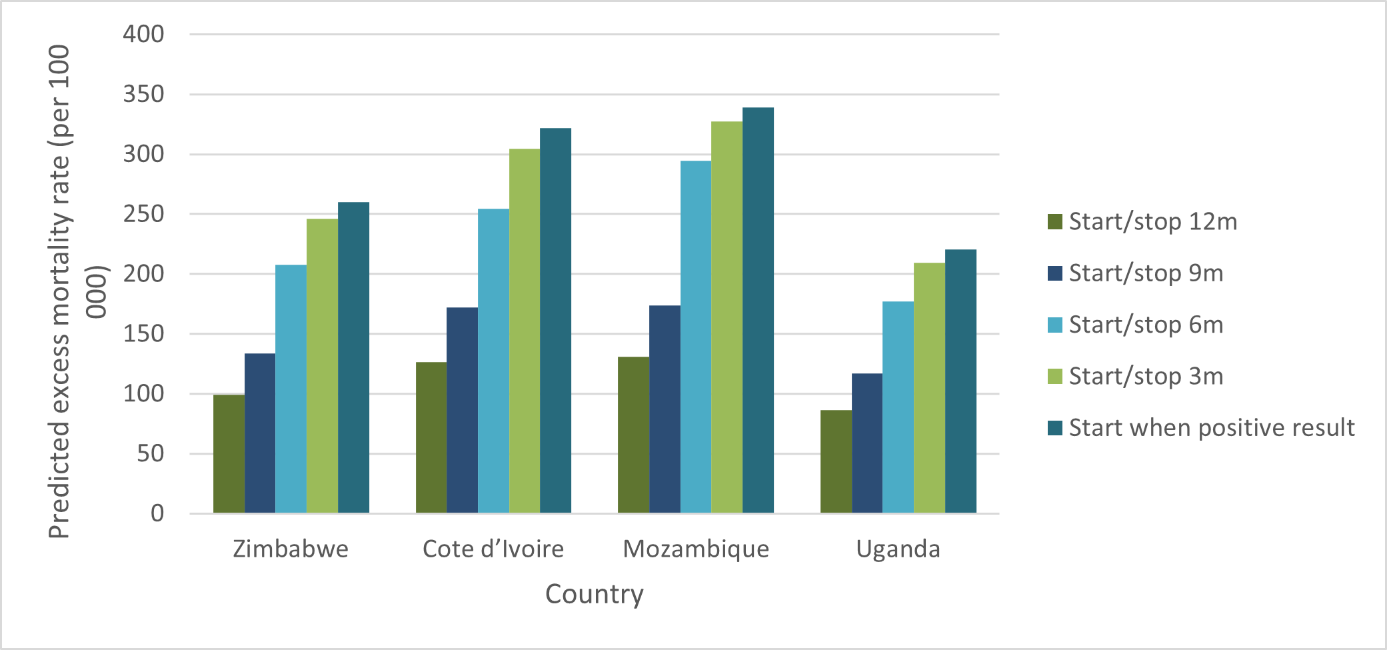


##

## **Fig C. Risk Ratios Under Alternative Cotrimoxazole Strategies.** Predicted risk ratio per year by country under alternative cotrimoxazole strategies.


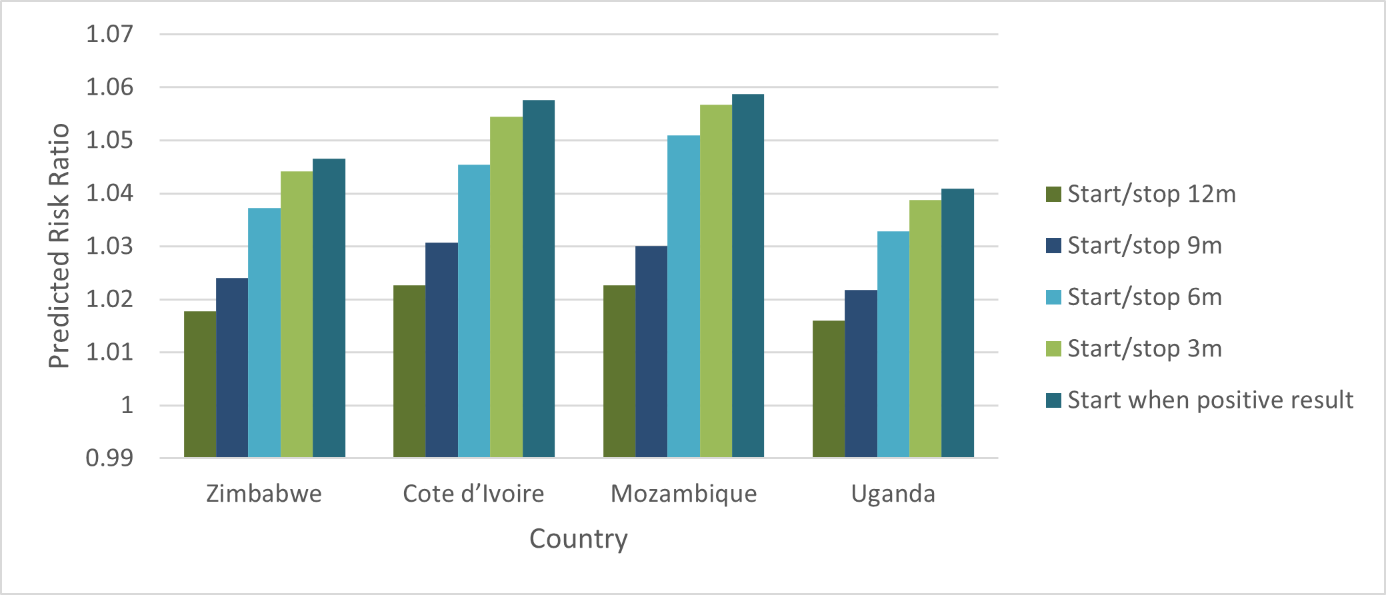


# Section 3: Combined Sensitivity Analysis

###

## **Fig D. Sensitivity Analysis for Zimbabwe (Risk Ratios).** Sensitivity analysis, for Zimbabwe, exploring the effect of varying assumptions on the Risk Ratio for deaths (6 weeks to 2 years) compared to the current WHO strategy


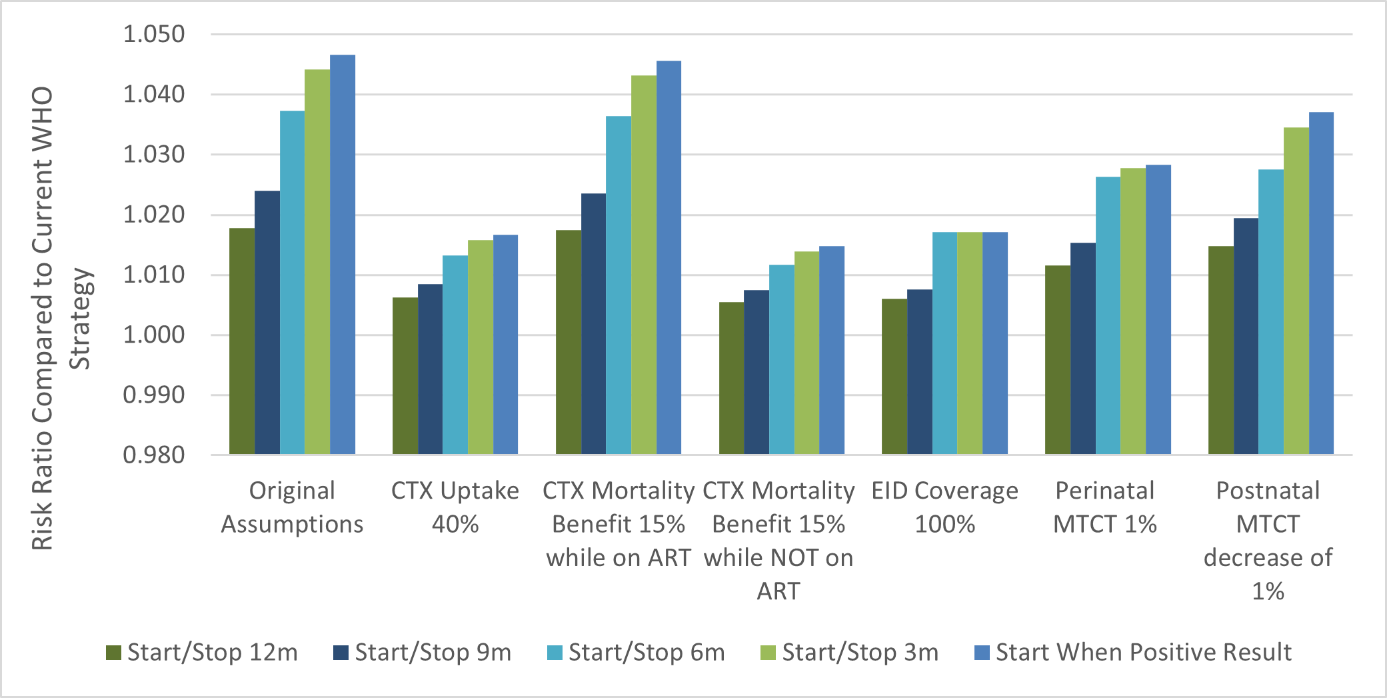


## **Fig E. Sensitivity Analysis for Zimbabwe (Excess Deaths).** Sensitivity analysis, for Zimbabwe, exploring the effect of varying assumptions on the Risk Ratio for deaths (6 weeks to 2 years) compared to the current WHO strategy


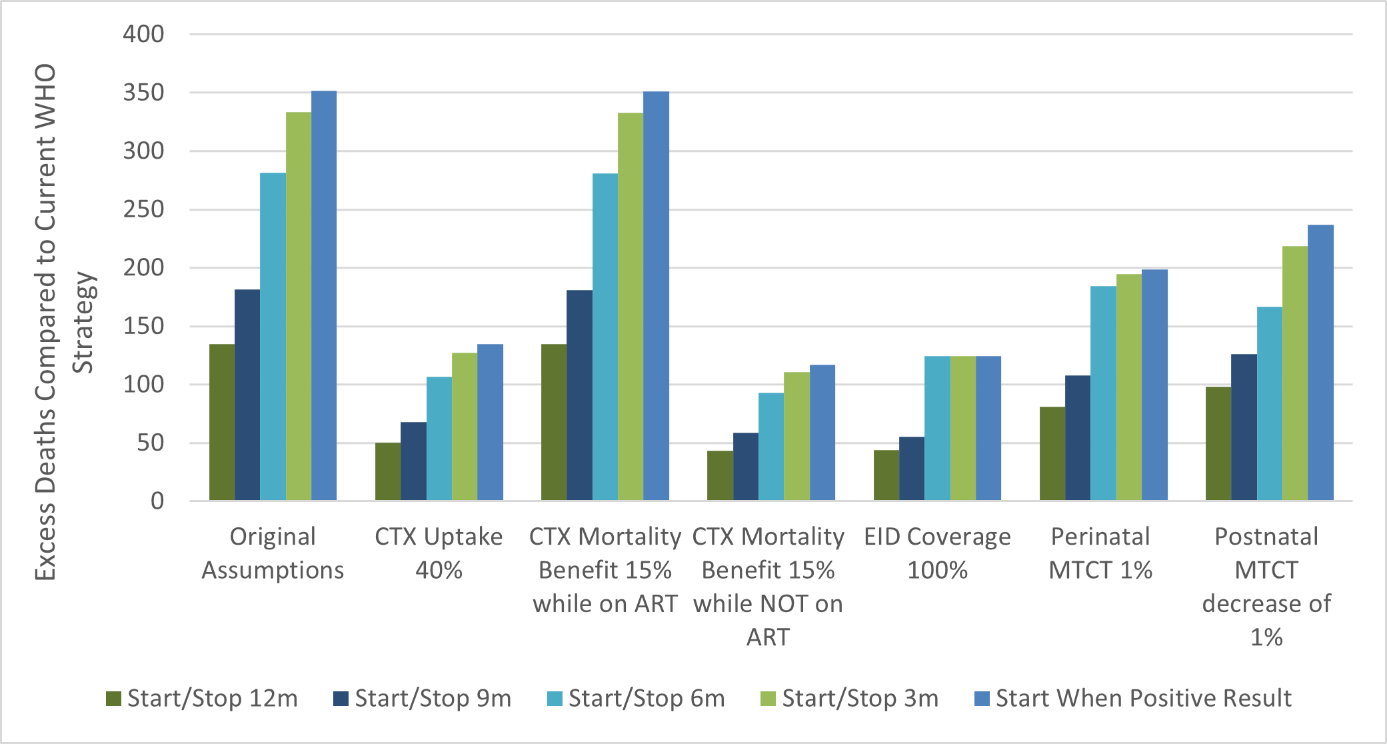


## **Fig F. Sensitivity Analysis for Cote d’Ivoire (Risk Ratios).** Sensitivity analysis, for Cote d’Ivoire, exploring the effect of varying assumptions on the Risk Ratio for deaths (6 weeks to 2 years) compared to the current WHO strategy


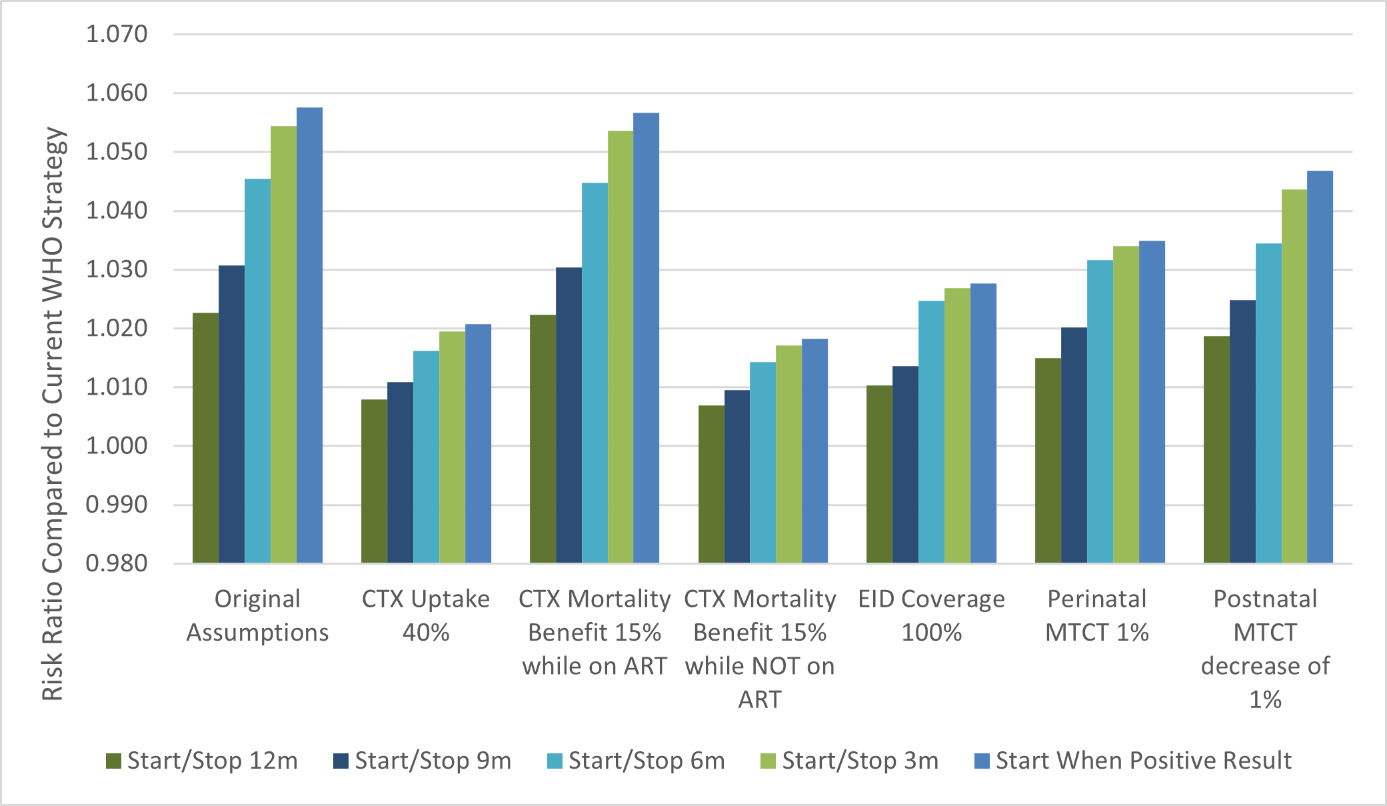


## **Fig G. Sensitivity Analysis for Cote d’Ivoire (Excess Deaths).** Sensitivity analysis, for Cote d’Ivoire, exploring the effect of varying assumptions on the Risk Ratio for deaths (6 weeks to 2 years) compared to the current WHO strategy


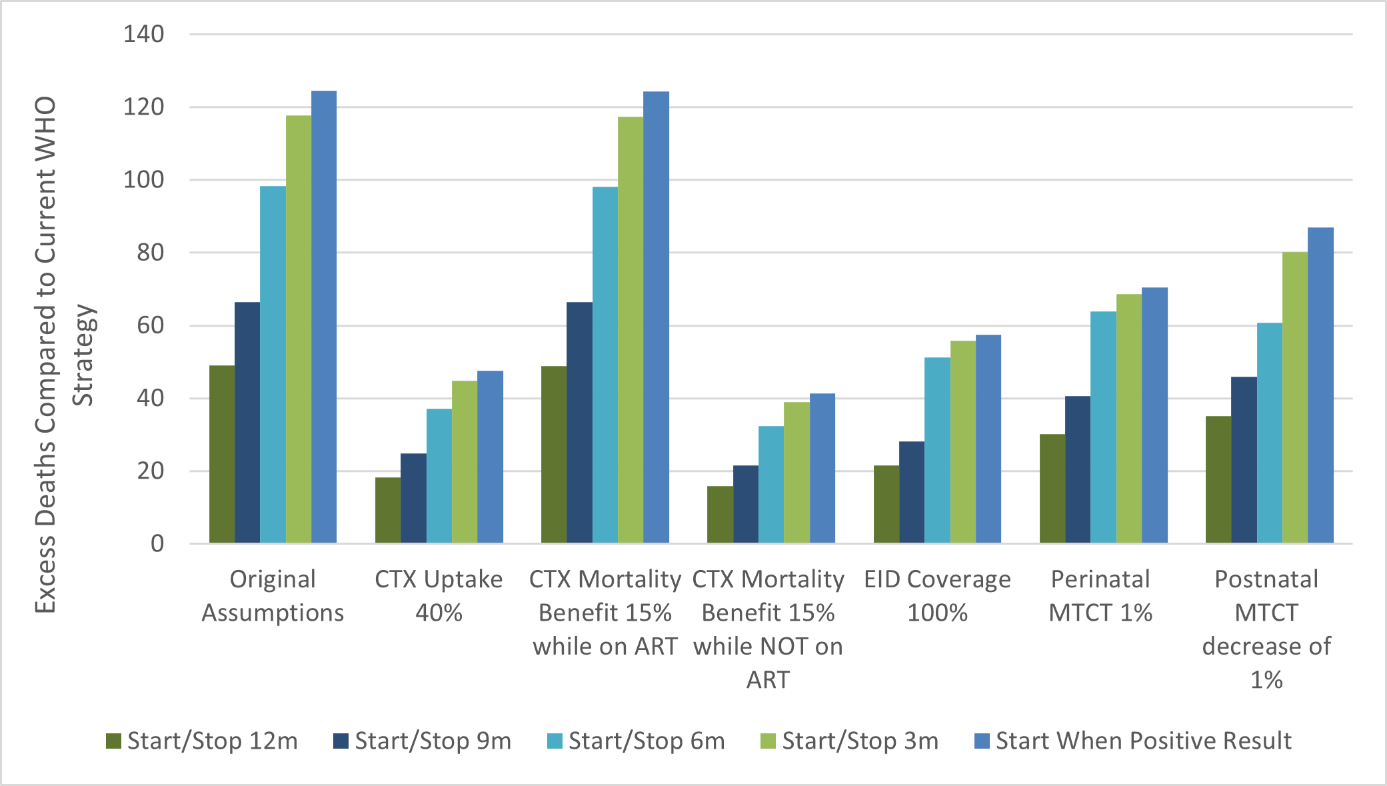


## **Fig H. Sensitivity Analysis for Mozambique (Risk Ratios).** Sensitivity analysis, for Mozambique, exploring the effect of varying assumptions on the Risk Ratio for deaths (6 weeks to 2 years) compared to the current WHO strategy


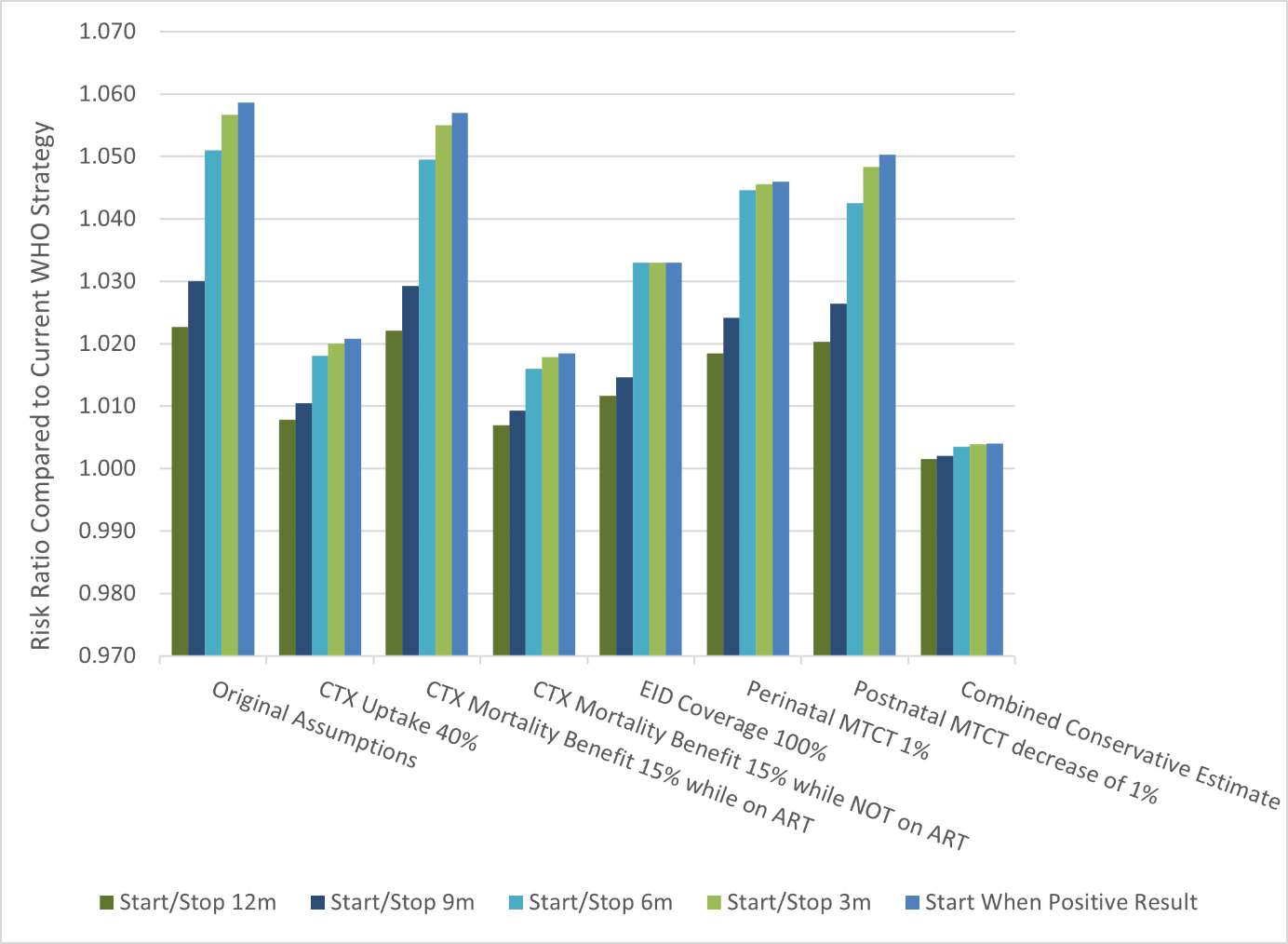


###

## **Fig I. Sensitivity Analysis for Uganda (Risk Ratios).** Sensitivity analysis, for Uganda, exploring the effect of varying assumptions on the Risk Ratio for deaths (6 weeks to 2 years) compared to the current WHO strategy


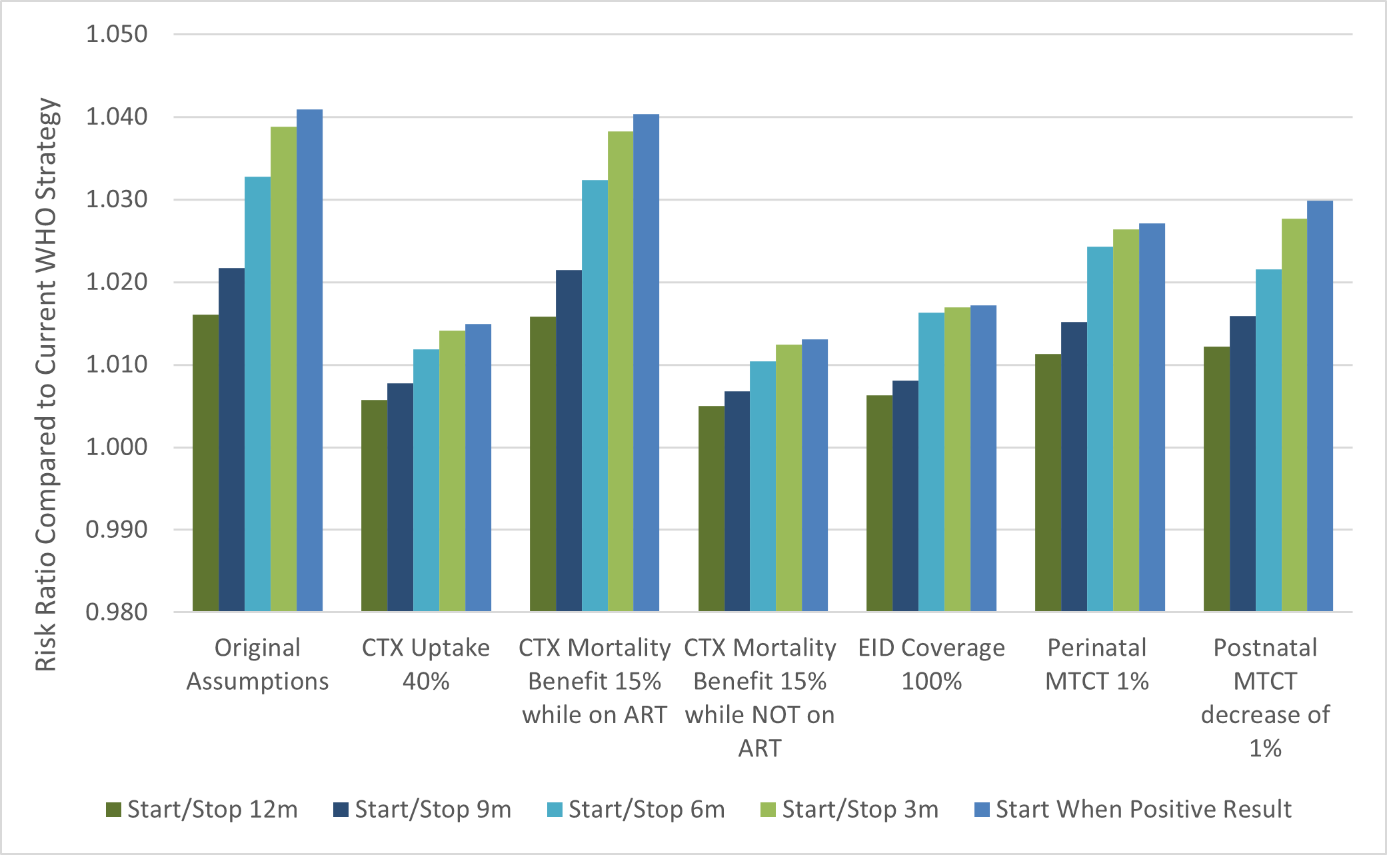


## **Fig J. Sensitivity Analysis for Uganda (Excess Deaths).** Sensitivity analysis, for Uganda, exploring the effect of varying assumptions on the Risk Ratio for deaths (6 weeks to 2 years) compared to the current WHO strategy


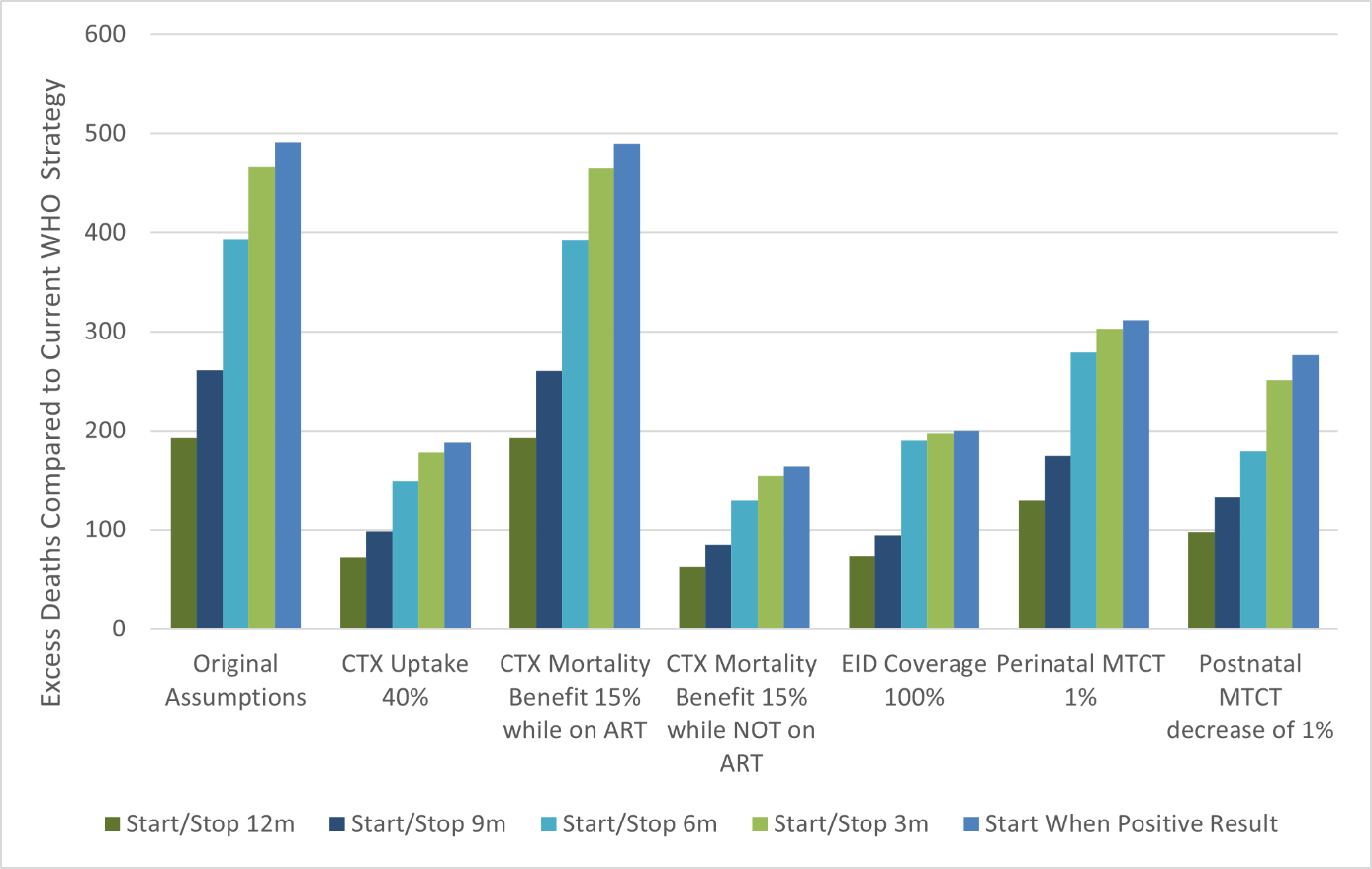


# Section 4: Sensitivity Analysis for Key Assumptions (Individual Variables)

## **Fig K. Sensitivity Variable - Cotrimoxazole Uptake, Zimbabwe.** Risk ratio of mortality for varying risk reduction from cotrimoxazole uptake for Zimbabwe, (Risk Ratio).


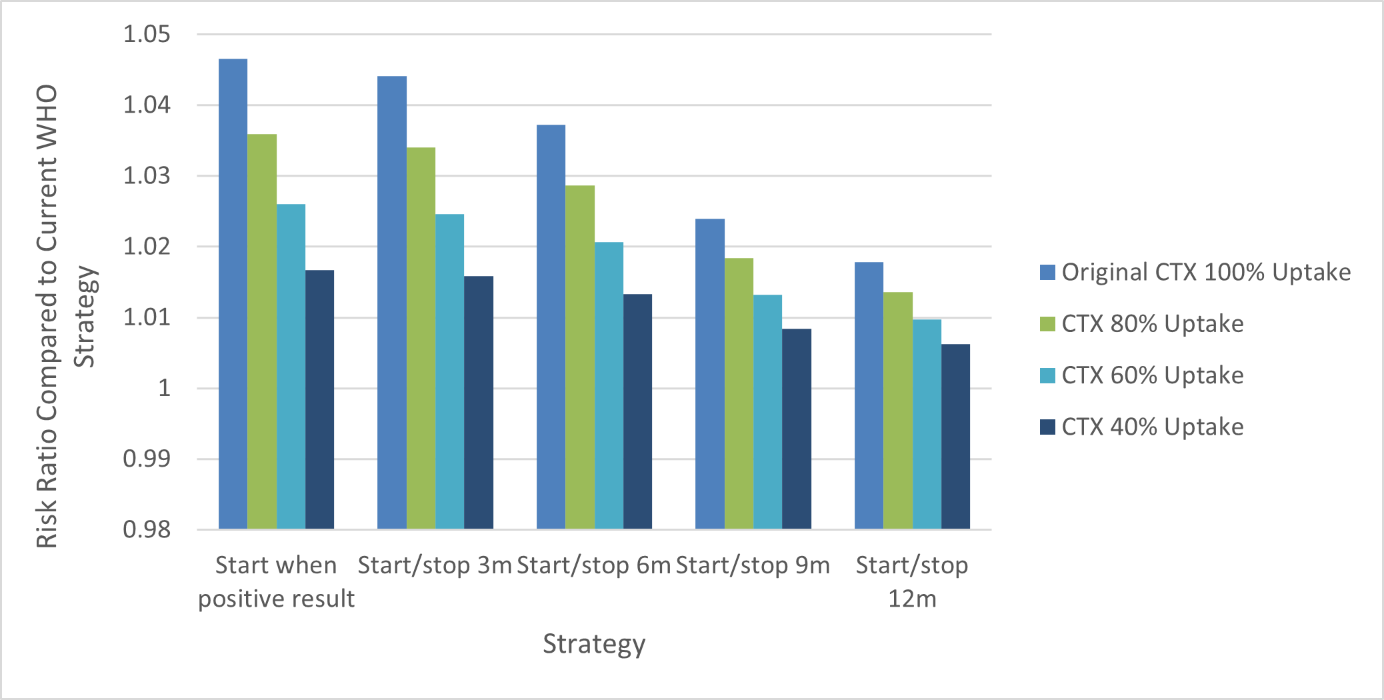


## **Fig L. Sensitivity Variable – Risk Reduction from CTX, Zimbabwe.** Risk ratio of mortality for varying risk reduction from cotrimoxazole while infant with HIV is taking antiretroviral therapy for Zimbabwe, (Risk Ratio). Risk reduction from 15%-43%.


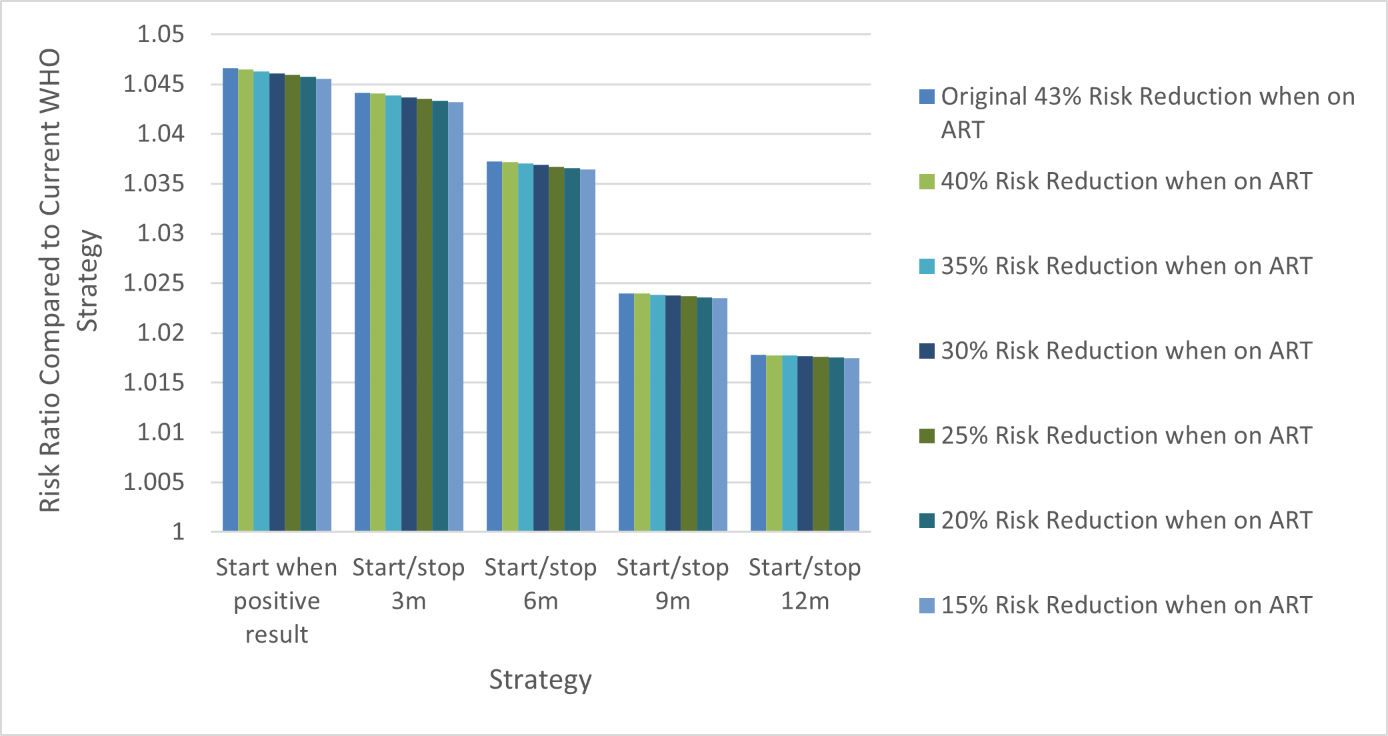


## **Fig M. Sensitivity Variable – Risk Reduction from CTX, Zimbabwe.** Risk ratio of mortality for varying risk reduction from cotrimoxazole while infant with HIV is taking antiretroviral therapy for Zimbabwe, (Risk Ratio). Risk Reduction from 25%-60%.


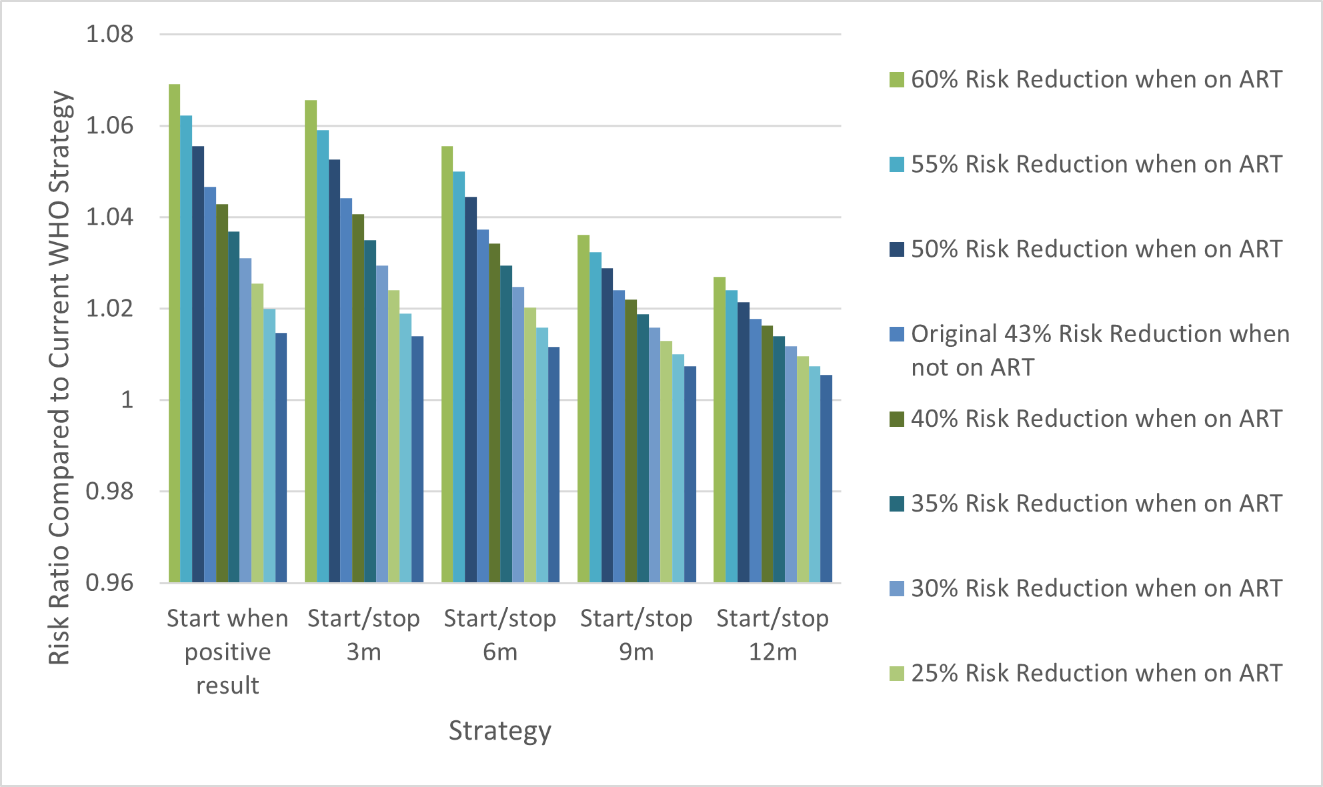


## **Fig N. Sensitivity Variable – EID Testing, Zimbabwe.** Risk ratio of mortality for varying probability of HIV-exposed infants undergoing Early Infant Diagnosis (EID) for Zimbabwe, (Risk Ratio).


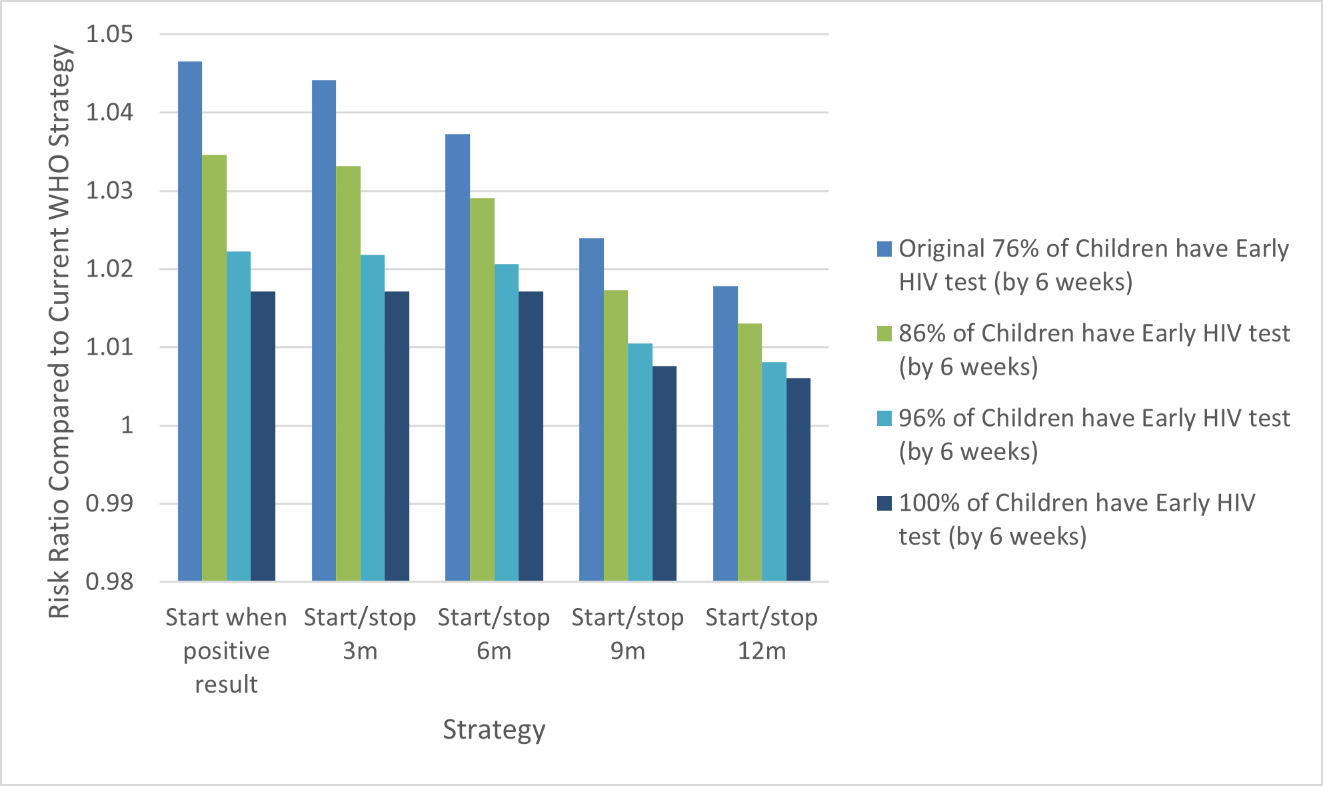


## **Fig O. Sensitivity Variable –** **Perinatal MTCT, Zimbabwe.** Risk ratio of mortality for varying probability of perinatal mother-to-child transmission (MTCT) for Zimbabwe, (Risk Ratio).


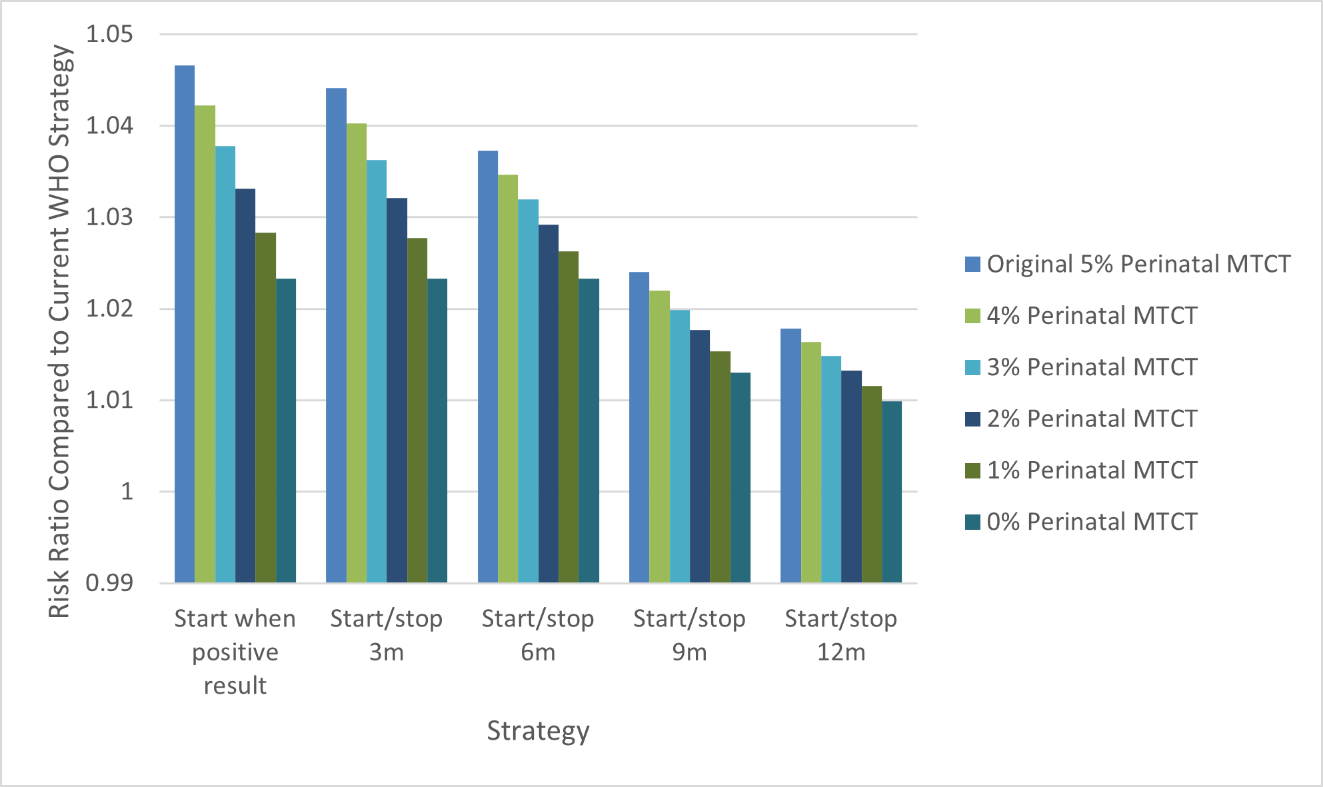


## **Fig P. Sensitivity Variable – Postnatal MTCT, Zimbabwe.** Risk ratio of mortality for varying probability of post-natal mother-to-child transmission (MTCT) for Zimbabwe, (Risk Ratio).


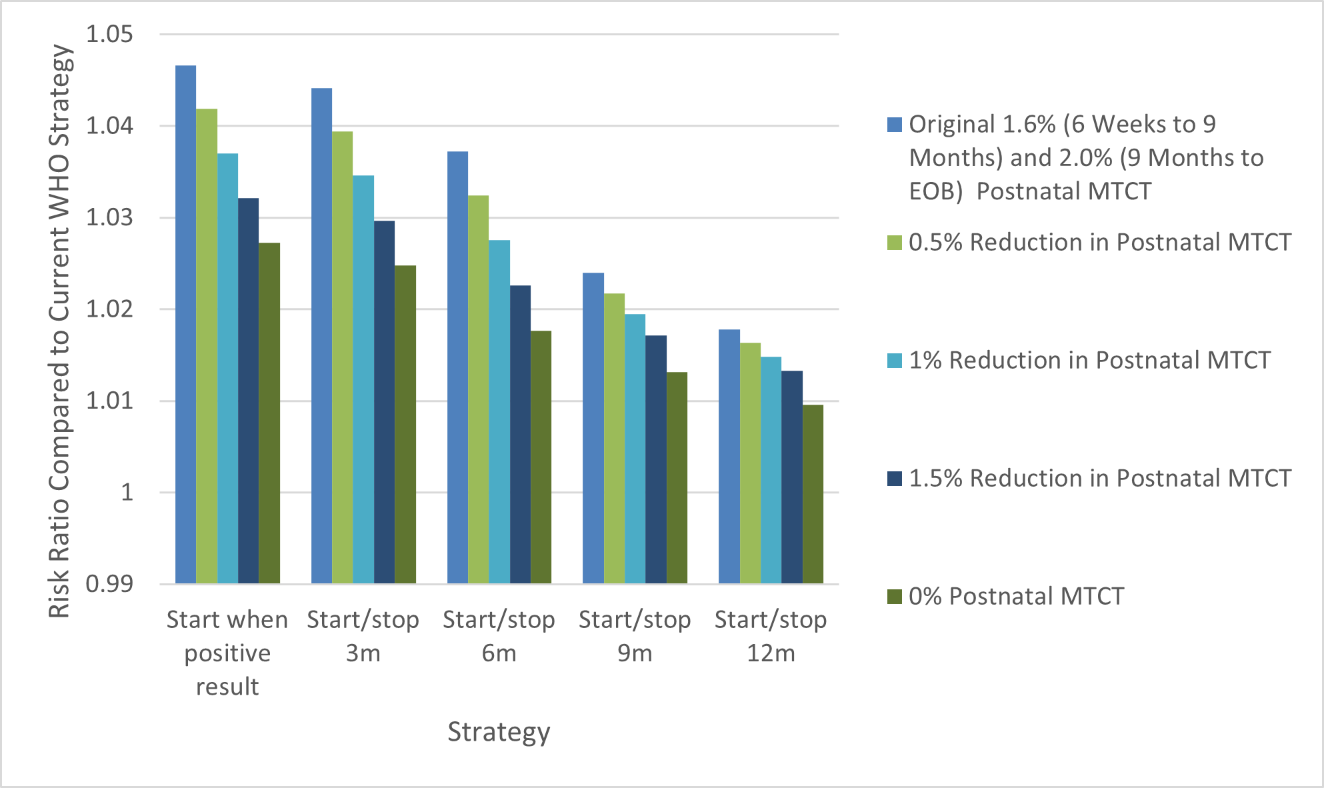


## **Fig Q. Sensitivity Variable - Cotrimoxazole Uptake, Cote d’Ivoire.** Risk ratio of mortality for varying risk reduction from cotrimoxazole uptake for Cote d’Ivoire, (Risk Ratio).


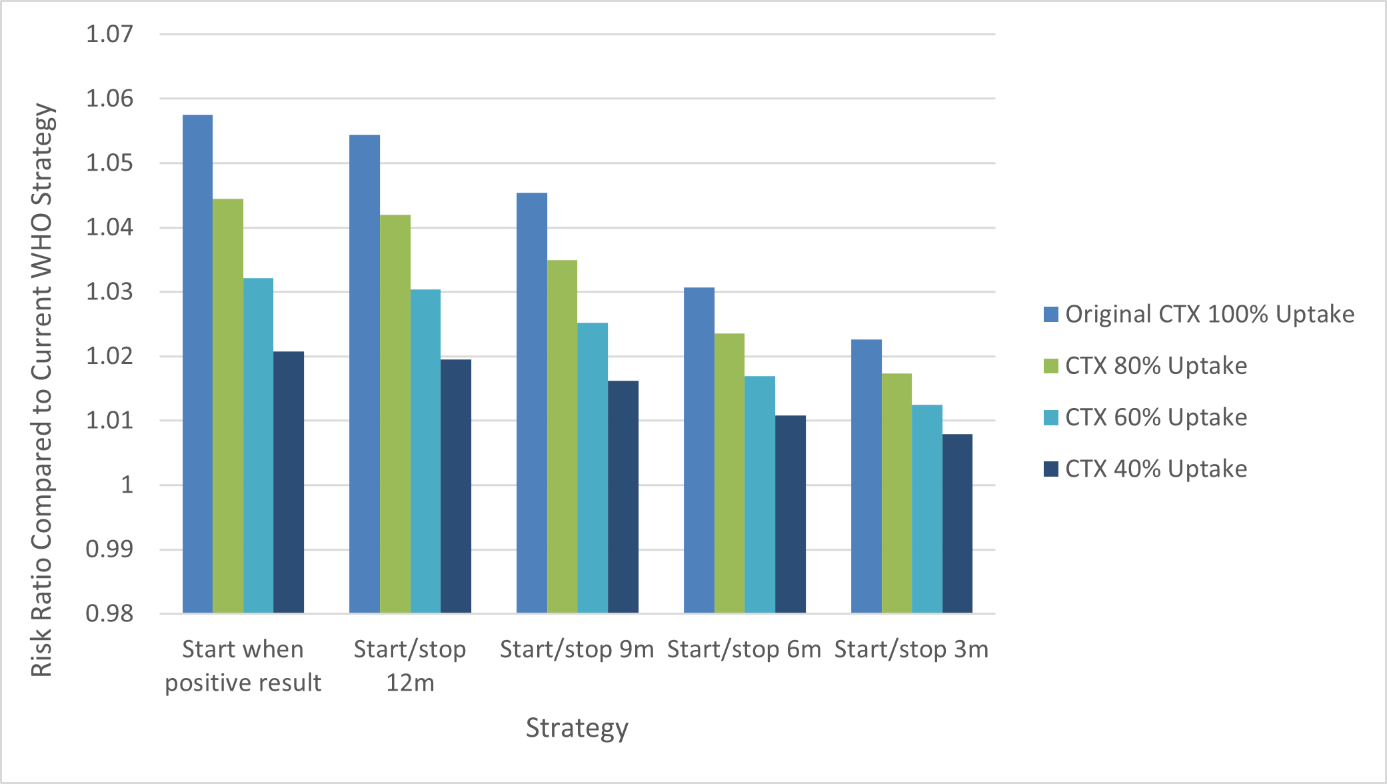


## **Fig R. Sensitivity Variable – Risk Reduction from CTX, Cote d’Ivoire.** Risk ratio of mortality for varying risk reduction from cotrimoxazole while infant with HIV is taking antiretroviral therapy for Cote d’Ivoire, (Risk Ratio). Risk reduction from 15%-43%.


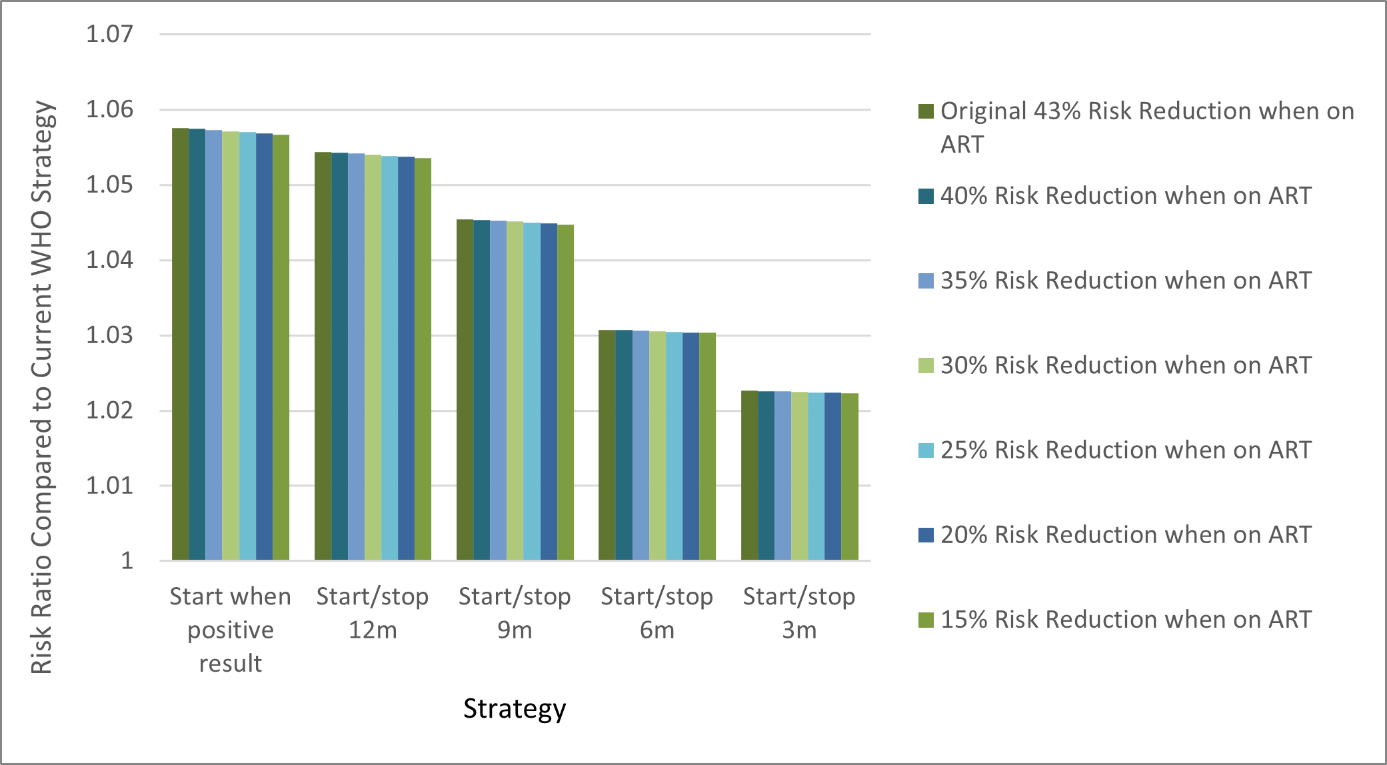


## **Fig S. Sensitivity Variable – Risk Reduction from CTX, Cote d’Ivoire.** Risk ratio of mortality for varying risk reduction from cotrimoxazole while infant with HIV is taking antiretroviral therapy for Cote d’Ivoire, (Risk Ratio). Risk reduction from 15%-60%.


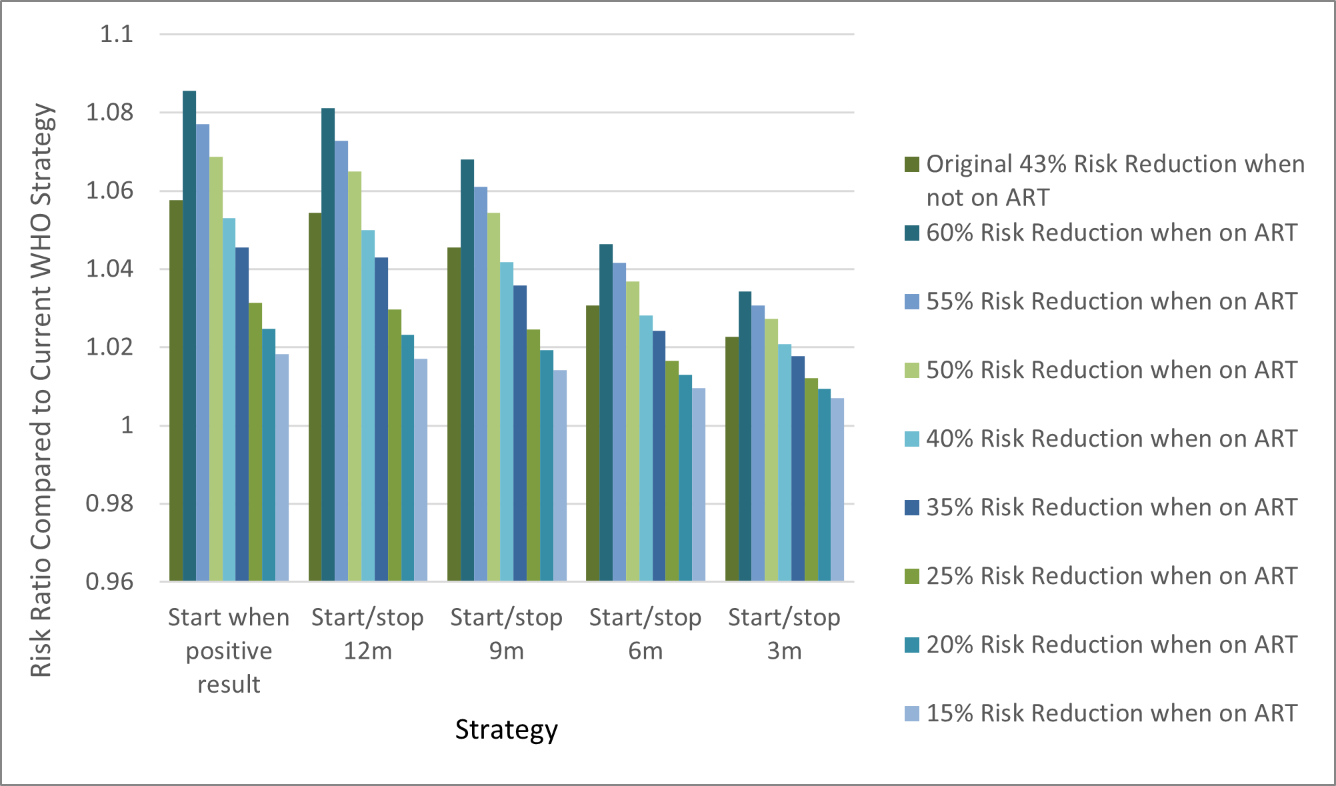


## **Fig T. Sensitivity Variable – EID Testing, Cote d’Ivoire.** Risk ratio of mortality for varying probability of HIV-exposed infants undergoing Early Infant Diagnosis (EID) for Cote d’Ivoire, (Risk Ratio).


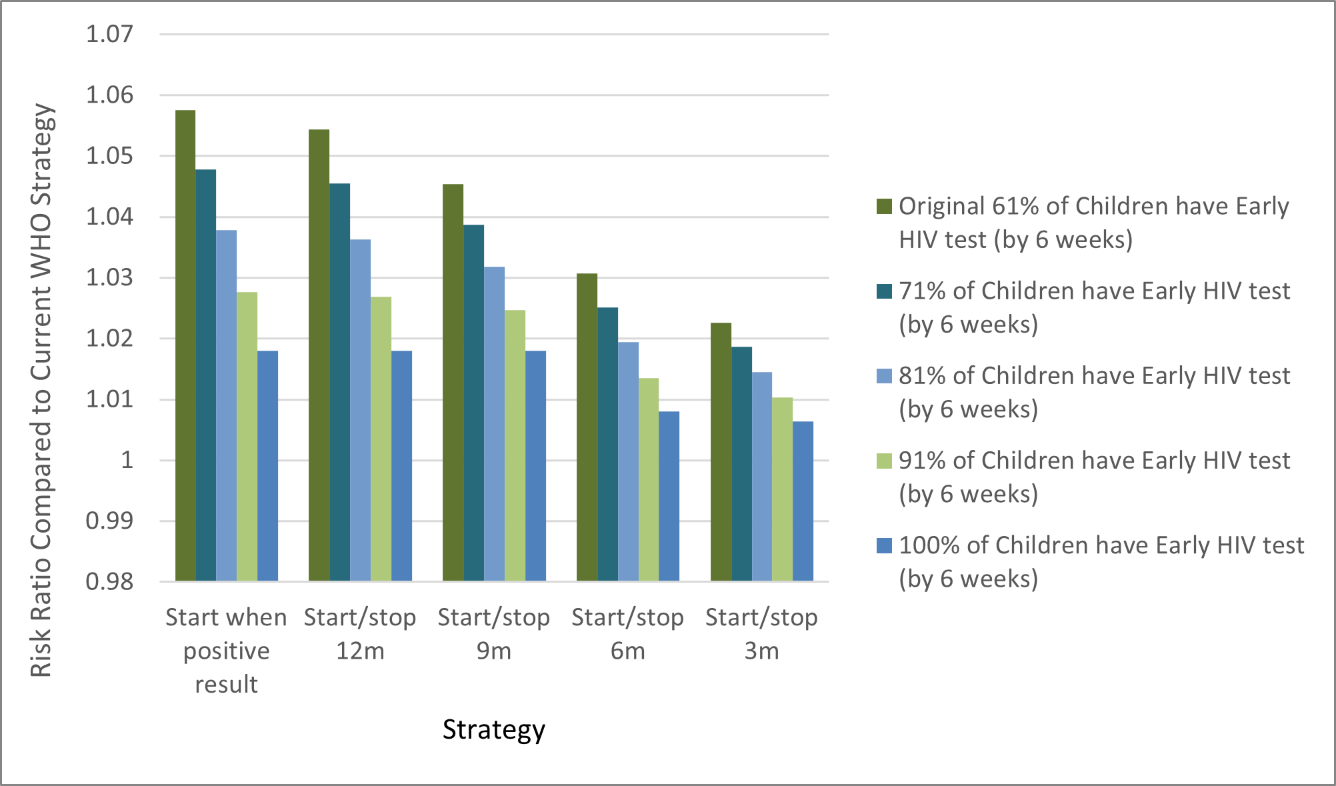


## **Fig U. Sensitivity Variable – Perinatal MTCT, Cote d’Ivoire.** Risk ratio of mortality for varying probability of perinatal mother-to-child transmission (MTCT) for Cote d’Ivoire, (Risk Ratio).


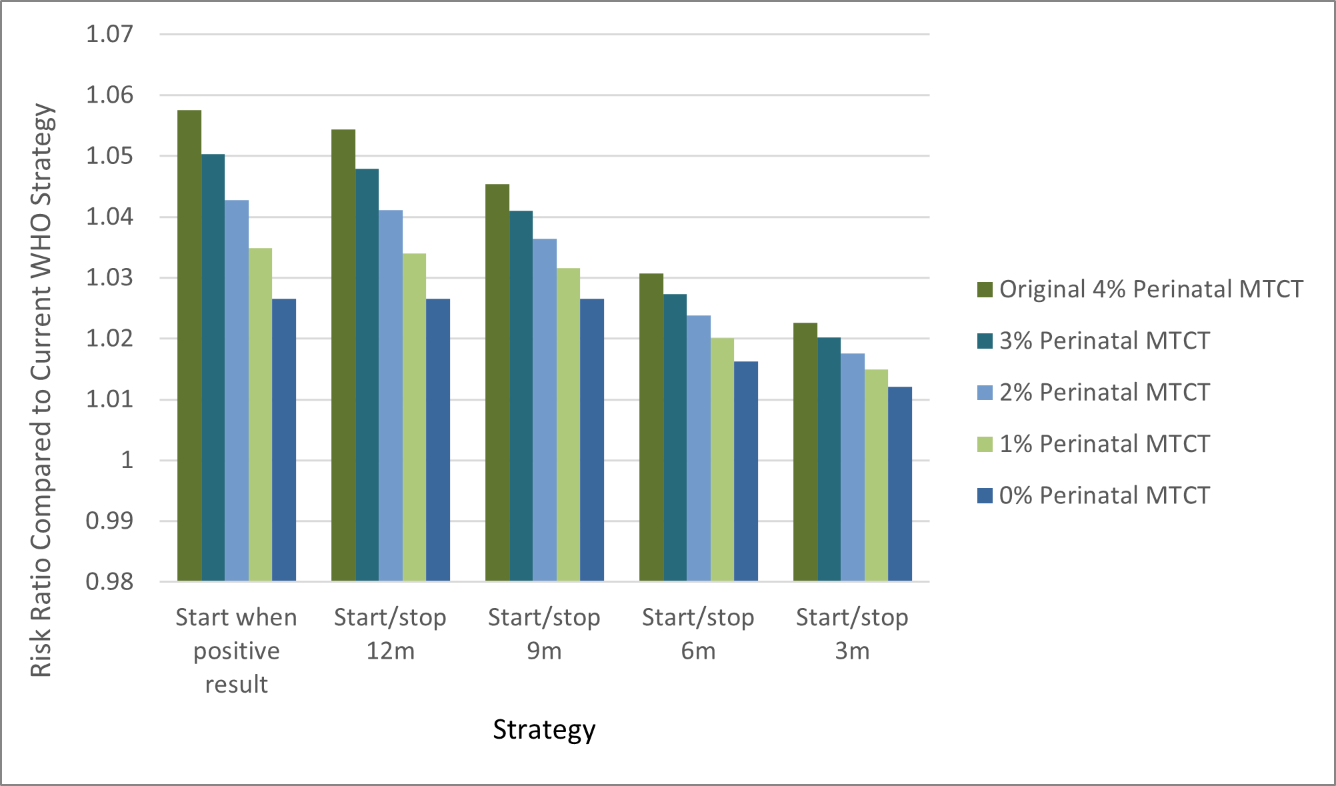


## **Fig V. Sensitivity Variable – Postnatal MTCT, Cote d’Ivoire.** Risk ratio of mortality for varying probability of post-natal mother-to-child transmission (MTCT) for Cote d’Ivoire, (Risk Ratio).


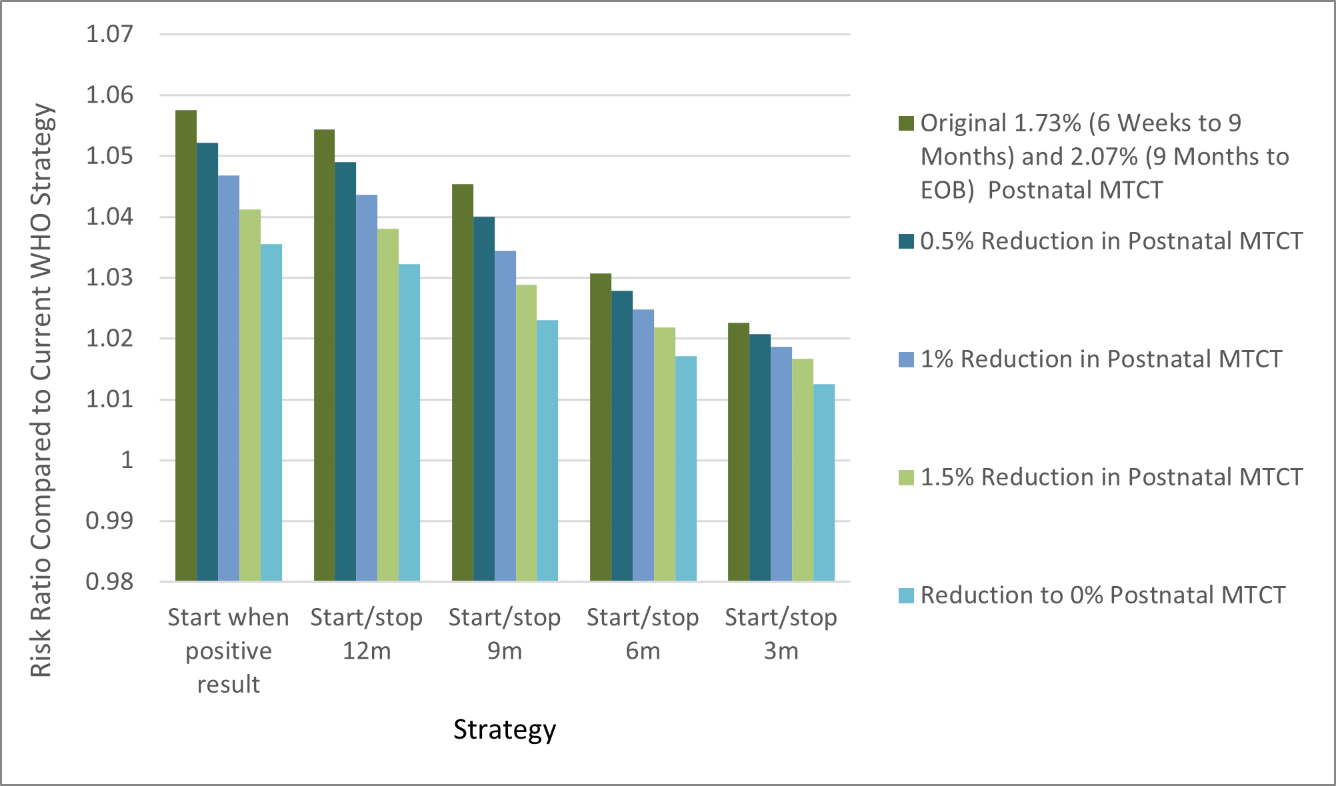


## **Fig W. Sensitivity Variable - Cotrimoxazole Uptake, Mozambique.** Risk ratio of mortality for varying risk reduction from cotrimoxazole uptake for Mozambique, (Risk Ratio).


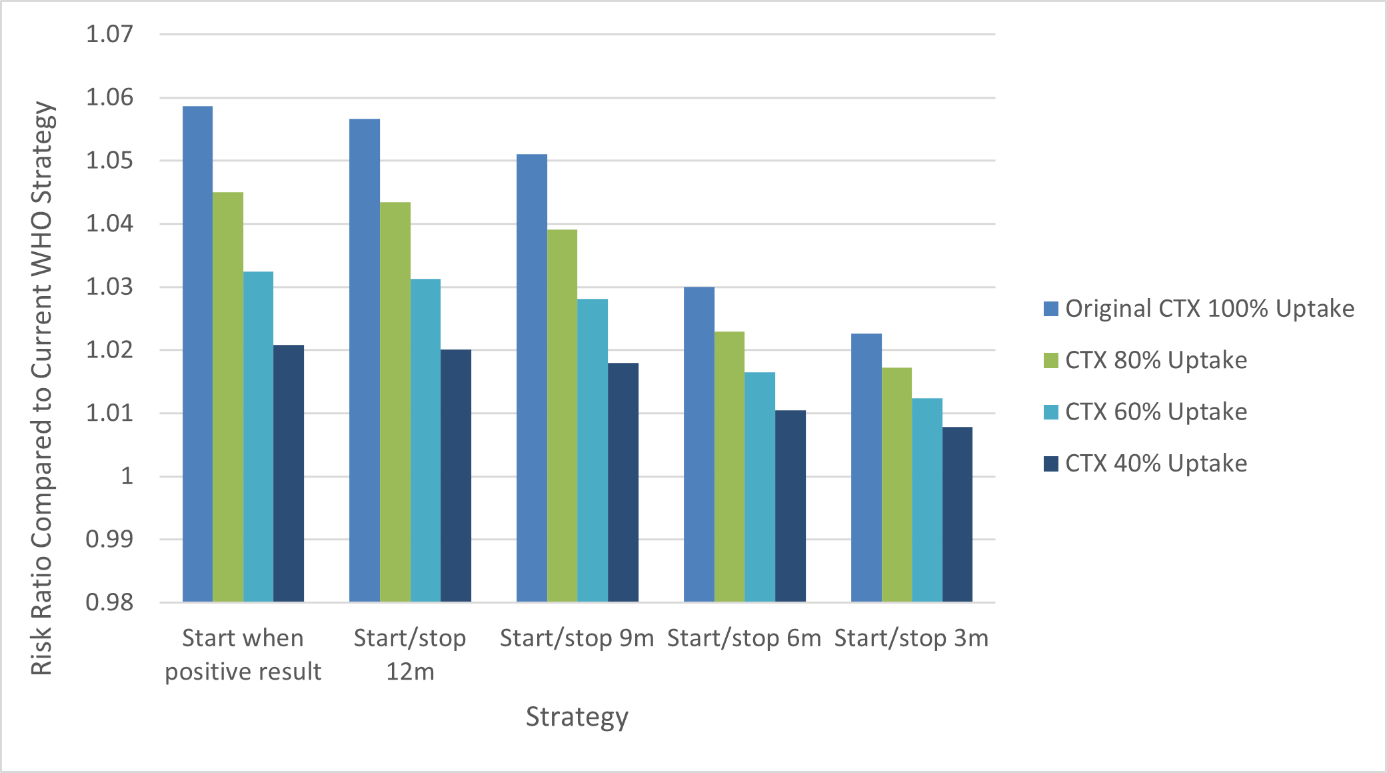


## **Fig X. Sensitivity Variable – Risk Reduction from CTX, Mozambique.** Risk ratio of mortality for varying risk reduction from cotrimoxazole while infant with HIV is taking antiretroviral therapy for Mozambique, (Risk Ratio). Risk reduction from 15%-43%.


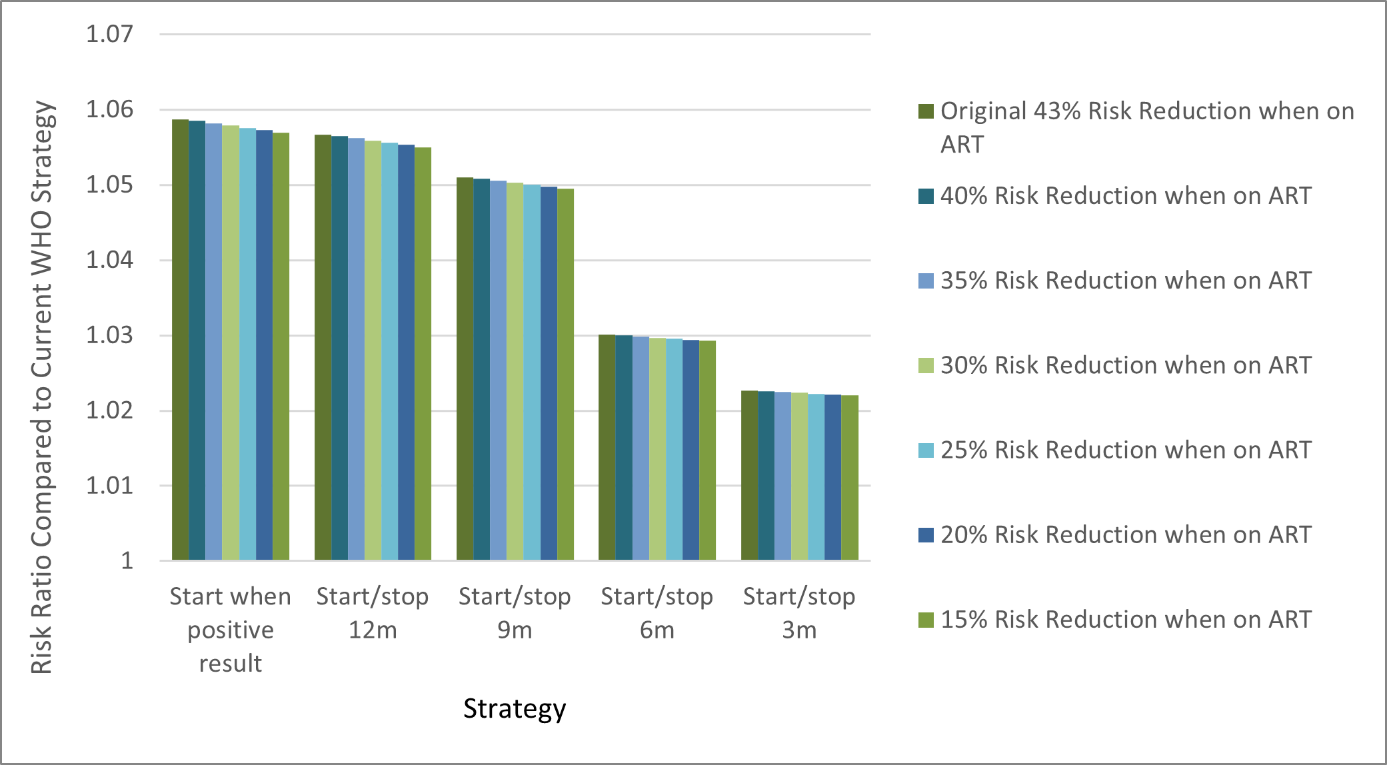


## **Fig Y. Sensitivity Variable – Risk Reduction from CTX, Mozambique.** Risk ratio of mortality for varying risk reduction from cotrimoxazole while infant with HIV is taking antiretroviral therapy for Mozambique, (Risk Ratio). Risk reduction from 15%-60%.


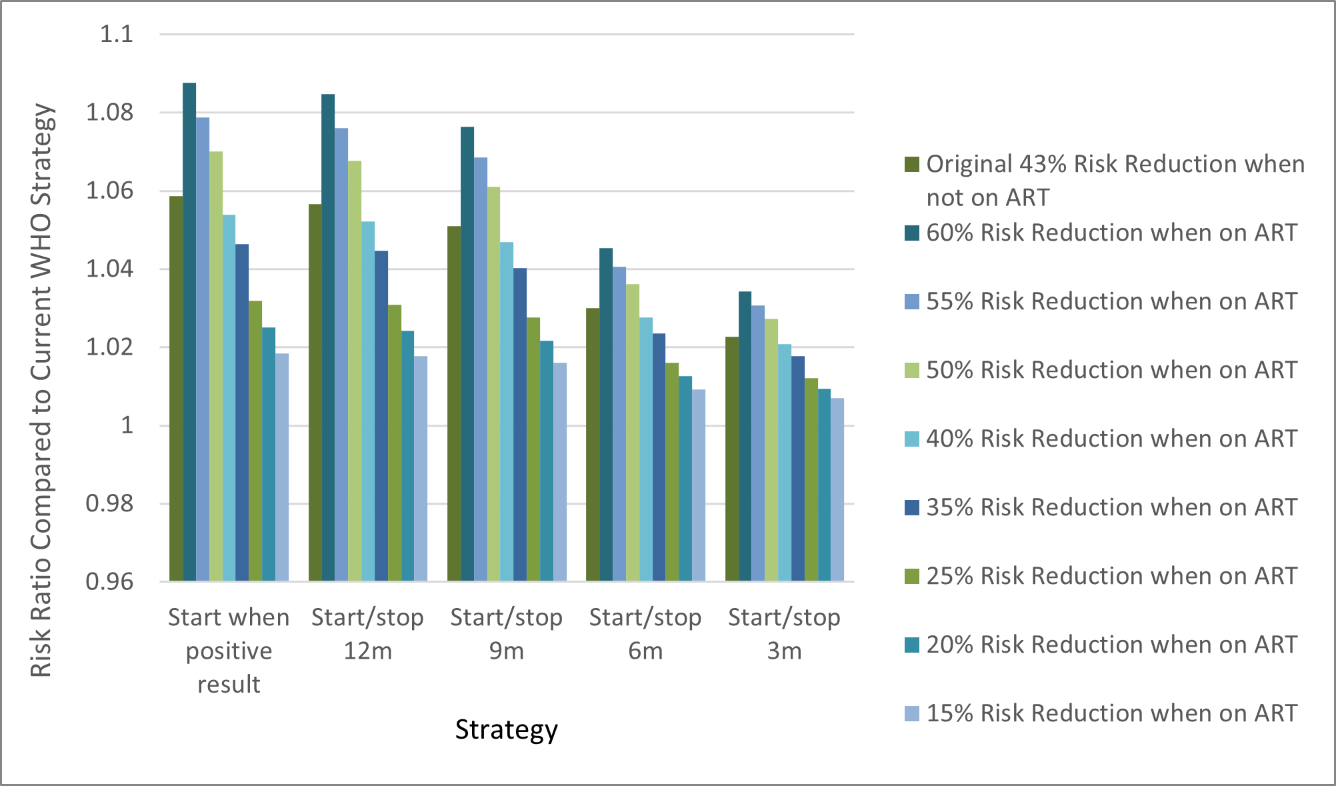


## **Fig Z. Sensitivity Variable – EID Testing, Mozambique.** Risk ratio of mortality for varying probability of HIV-exposed infants undergoing Early Infant Diagnosis (EID) for Mozambique, (Risk Ratio).


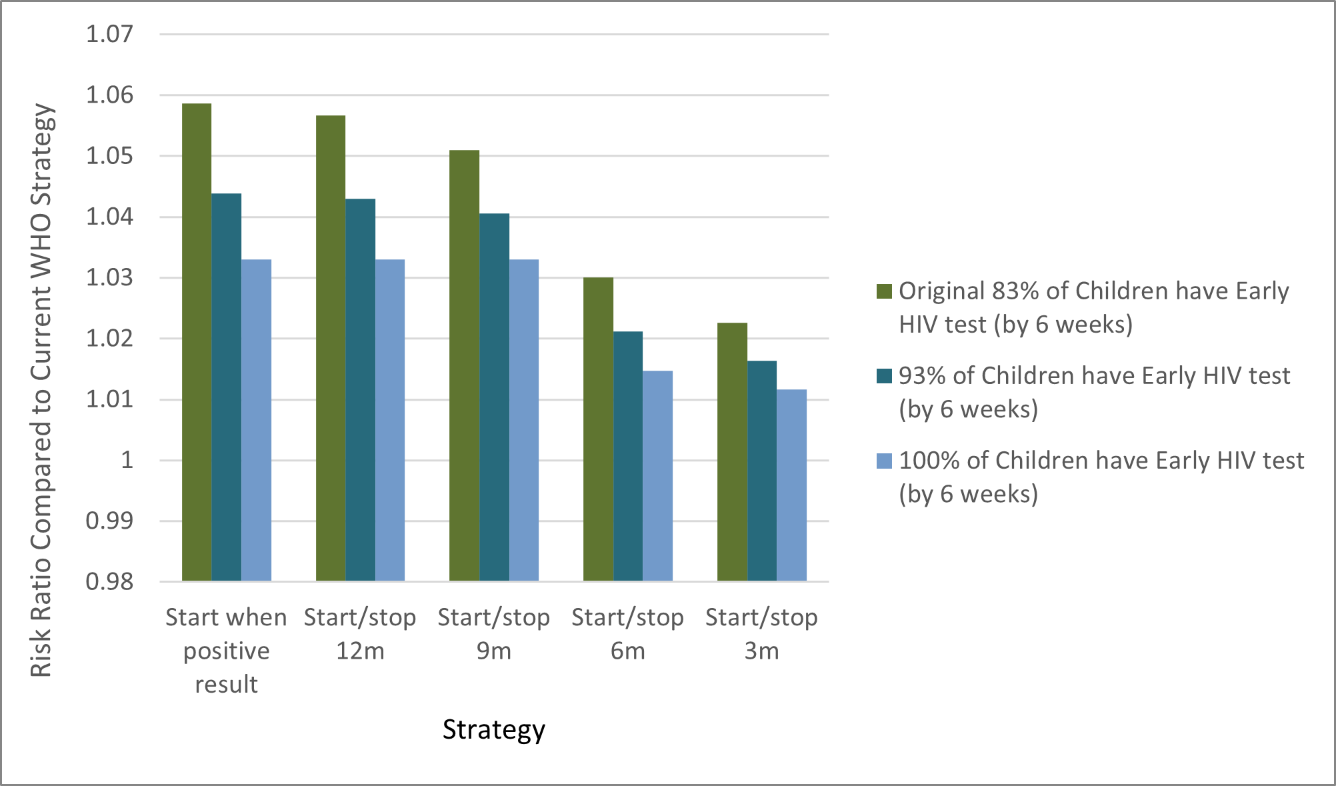


## **Fig AA. Sensitivity Variable – Perinatal MTCT, Mozambique.** Risk ratio of mortality for varying probability of perinatal mother-to-child transmission (MTCT) for Mozambique, (Risk Ratio).


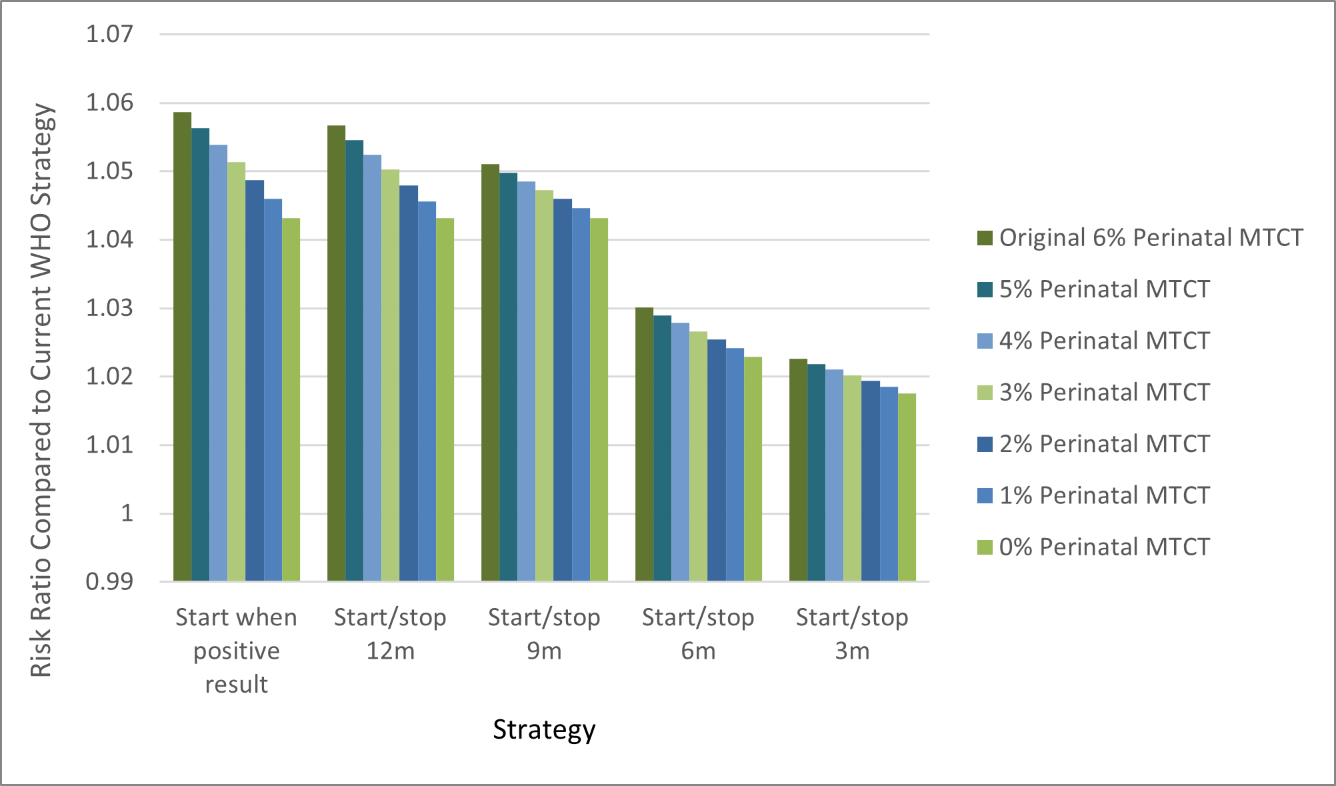


## **Fig AB. Sensitivity Variable – Postnatal MTCT, Mozambique.** Risk ratio of mortality for varying probability of post-natal mother-to-child transmission (MTCT) for Mozambique, (Risk Ratio).


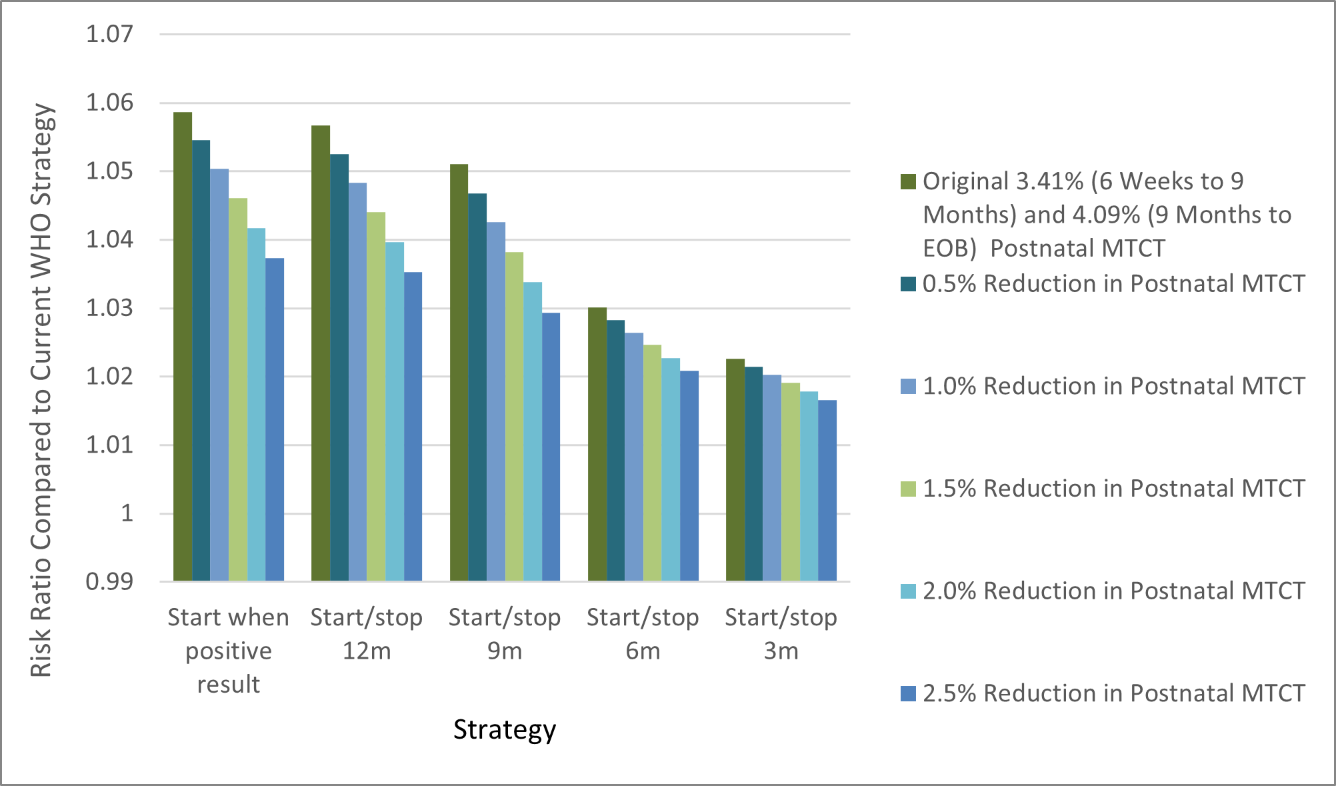


## **Fig AC. Sensitivity Variable - Cotrimoxazole Uptake, Uganda.** Risk ratio of mortality for varying risk reduction from cotrimoxazole uptake for Uganda, (Risk Ratio).


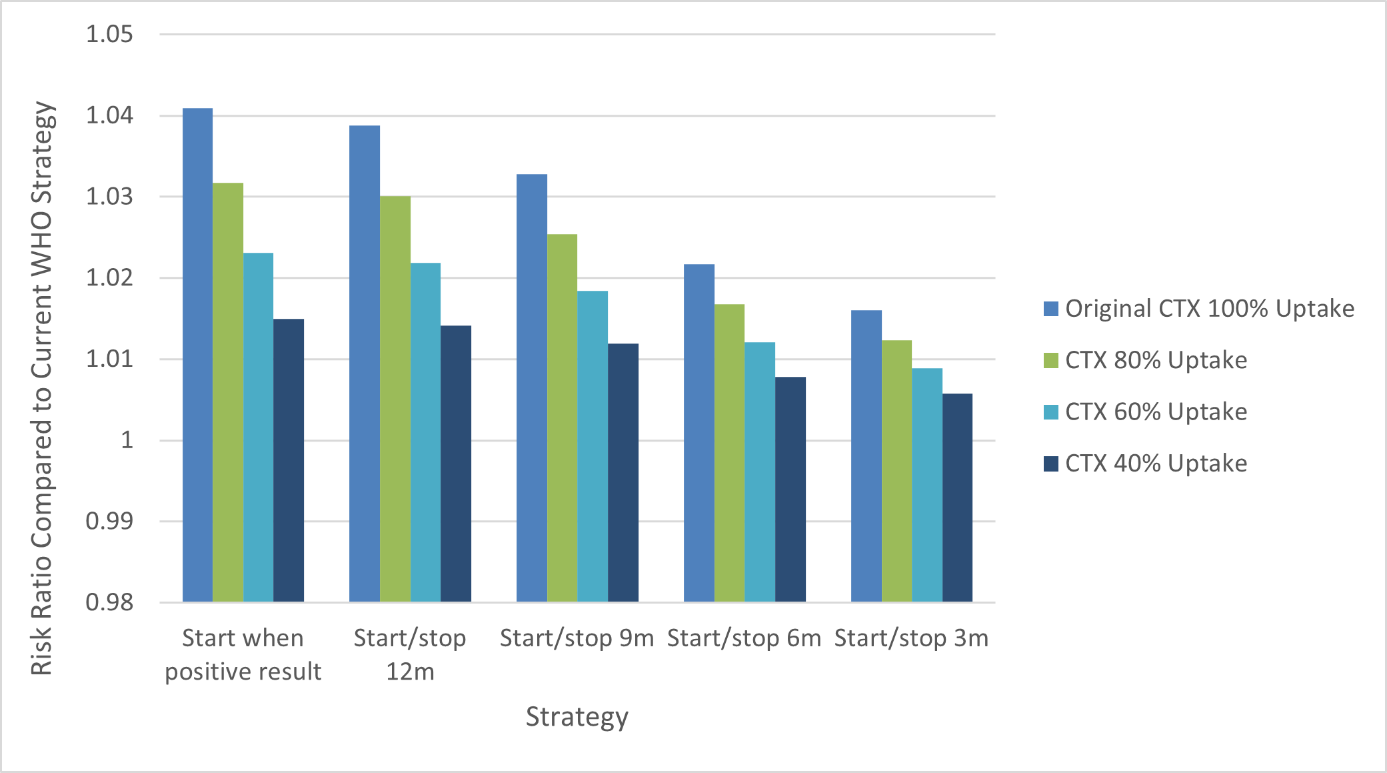


## **Fig AD. Sensitivity Variable – Risk Reduction from CTX, Uganda.** Risk ratio of mortality for varying risk reduction from cotrimoxazole while infant with HIV is taking antiretroviral therapy for Uganda, (Risk Ratio). Risk reduction from 15%-43%


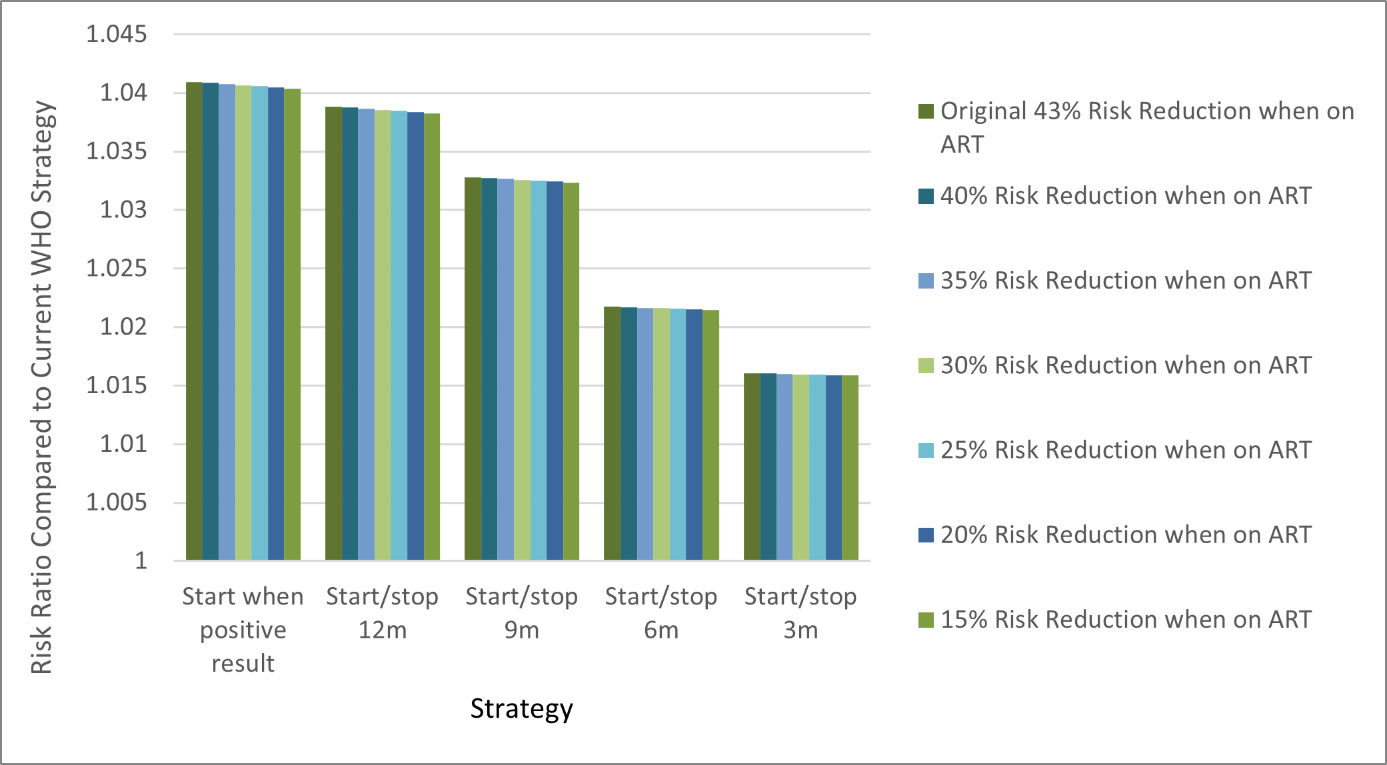


## **Fig AE. Sensitivity Variable – Risk Reduction from CTX, Uganda.** Risk ratio of mortality for varying risk reduction from cotrimoxazole while infant with HIV is taking antiretroviral therapy for Uganda, (Risk Ratio). Risk reduction from 15%-60%.


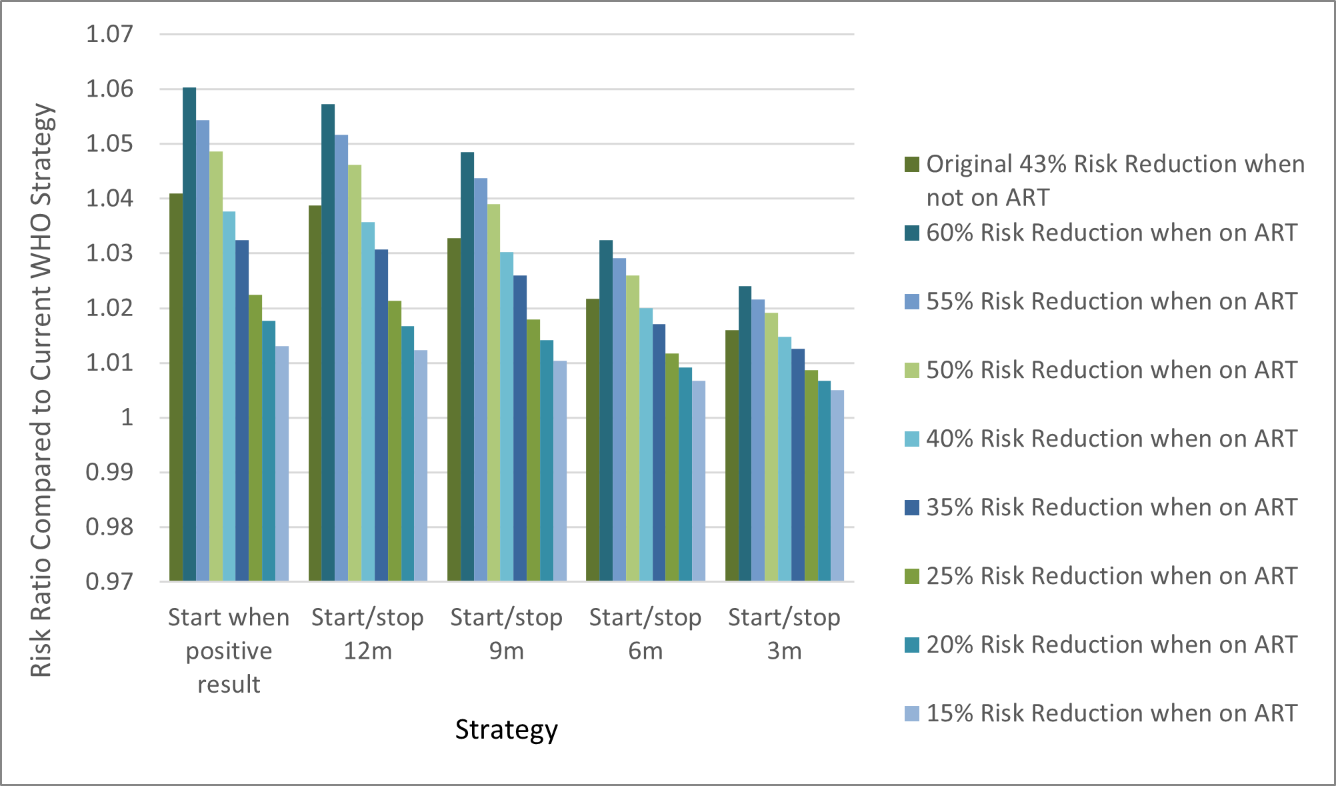


## **Fig AF. Sensitivity Variable – EID Testing, Uganda.** Risk ratio of mortality for varying probability of HIV-exposed infants undergoing Early Infant Diagnosis (EID) for Uganda, (Risk Ratio).


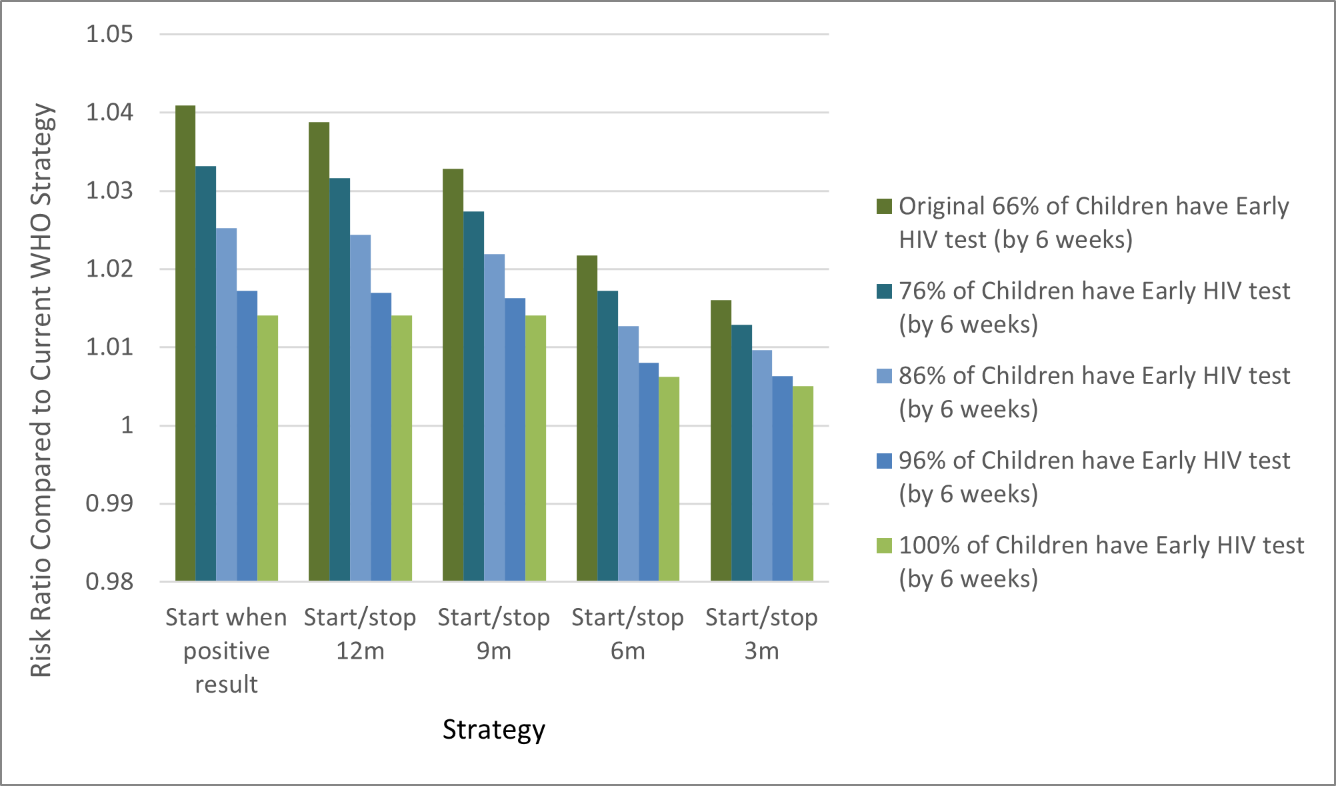


## **Fig AG. Sensitivity Variable – Perinatal MTCT, Uganda.** Risk ratio of mortality for varying probability of perinatal mother-to-child transmission (MTCT) for Uganda, (Risk Ratio).


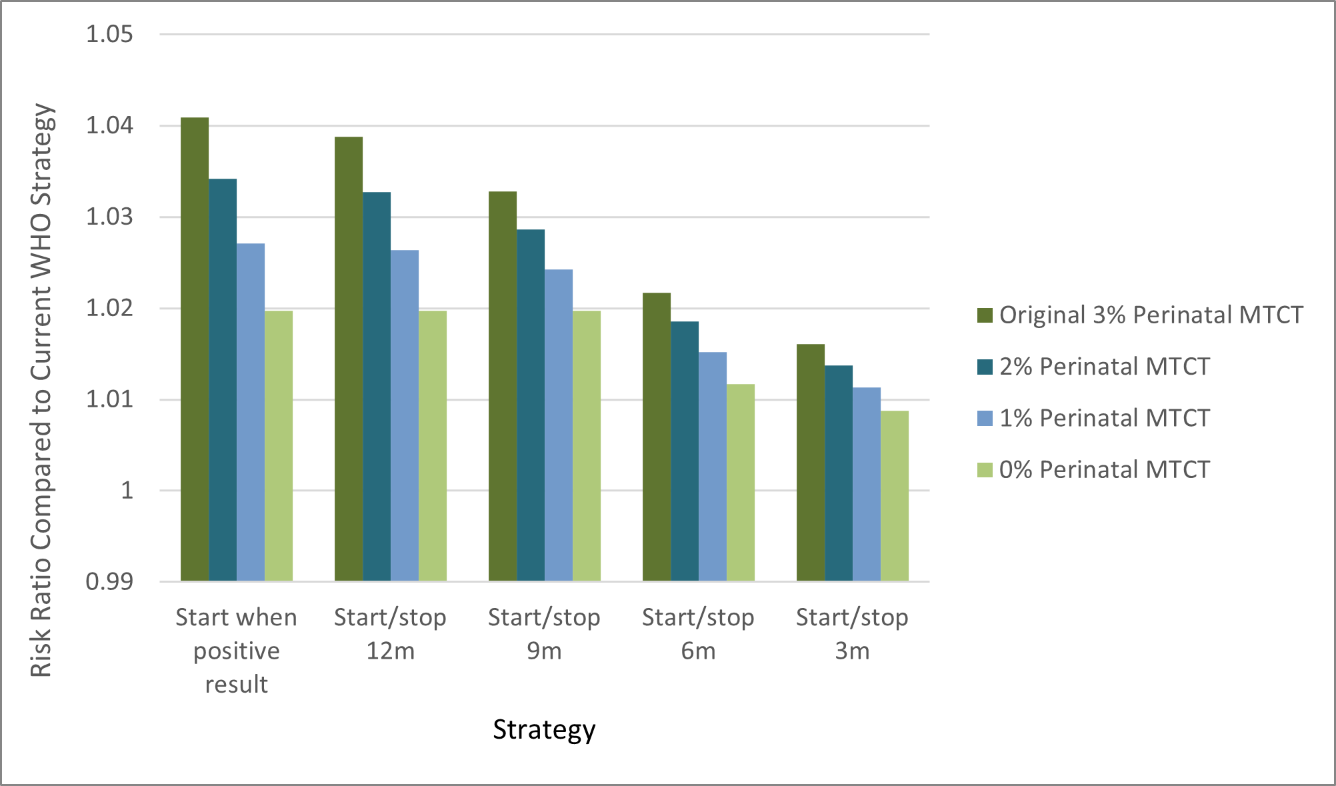


## **Fig AH. Sensitivity Variable – Postnatal MTCT, Uganda.** Risk ratio of mortality for varying probability of post-natal mother-to-child transmission (MTCT) for Uganda, (Risk Ratio).


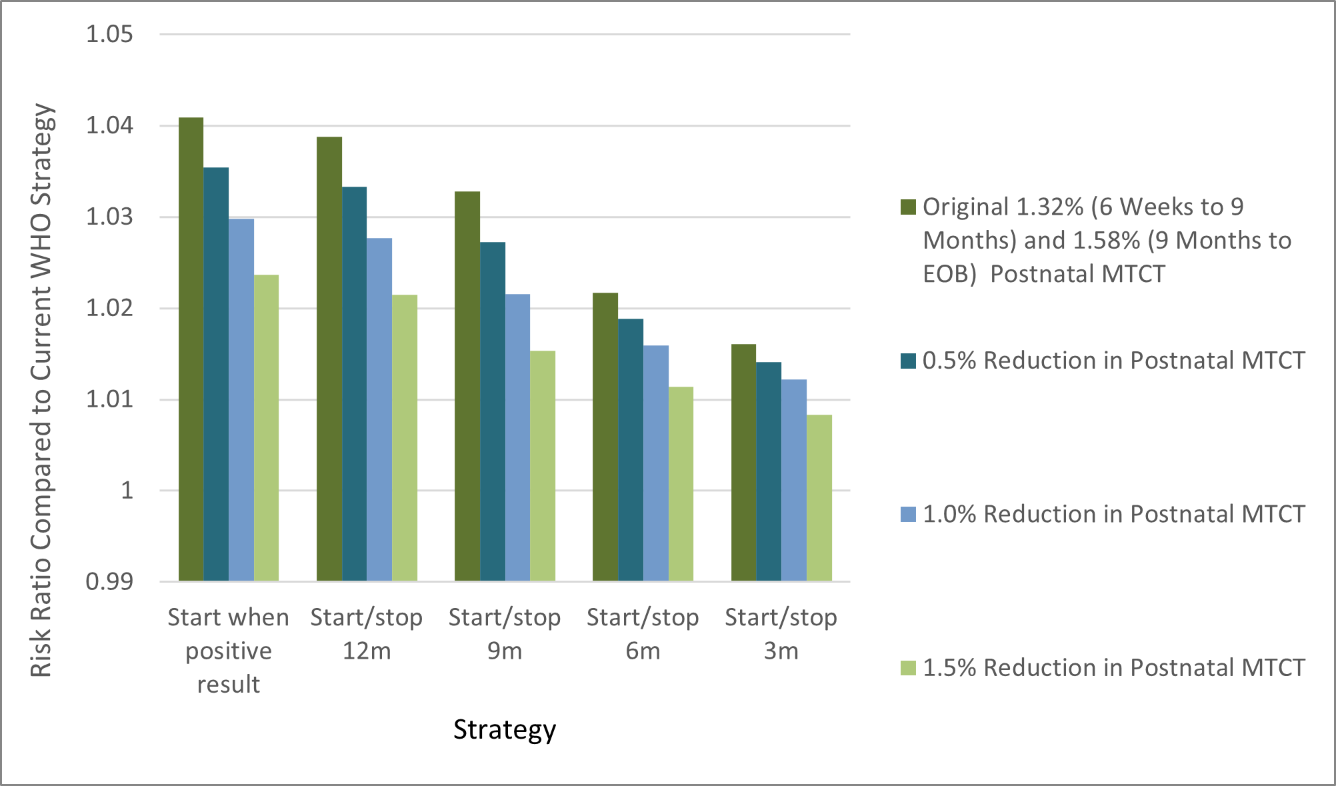

Supplement: S1 Text — Table A in S1 Text. Model assumptions and data sources. CTX = cotrimoxazole, ART = antiretroviral therapy, EID = early infant diagnosis at 6 weeks, ZIM = Zimbabwe, CIV = Cote d’Ivoire, MOZ = Mozambique, UGA = Uganda. *The authorship brings multidisciplinary expertise in paediatric HIV (AJP, CE, DMG, CW, MP), epidemiology (MS, DMG), clinical trials (DMG, AJP, MP, MS), and policy (SM, MP). Fig A in S1 Text. Predicted mortality percentage per year by country under alternative cotrimoxazole strategies. Columns represent additional deaths from each alternate strategy in comparison to the current WHO programmatic strategy of providing cotrimoxazole to all HIV-exposed infants. 12m = 12 months, 9m = 9 months, 6m = 6 months, 3m = 3 months. Fig B in S1 Text. Predicted excess mortality rate (per 100,000) per year by country under alternative cotrimoxazole strategies. Columns represent additional deaths from each alternate strategy in comparison to the current WHO programmatic strategy of providing cotrimoxazole to all HIV-exposed infants. 12m = 12 months, 9m = 9 months, 6m = 6 months, 3m = 3 months. Fig C in S1 Text. Predicted risk ratio per year by country under alternative cotrimoxazole strategies. Columns represent additional deaths from each alternate strategy in comparison to the current WHO programmatic strategy of providing cotrimoxazole to all HIV-exposed infants. 12m = 12 months, 9m = 9 months, 6m = 6 months, 3m = 3 months. Fig D in S1 Text. Sensitivity Analysis for Zimbabwe (risk ratios). Sensitivity analysis, for Zimbabwe, exploring the effect of varying assumptions on the risk ratio for deaths (6 weeks to 2 years) compared to the current WHO strategy. CTX = cotrimoxazole, ART = antiretroviral therapy, EID = early infant diagnosis at 6 weeks, MTCT = mother-to-child transmission, 12m = 12 months, 9m = 9 months, 6m = 6 months, 3m = 3 months. Fig E in S1 Text. Sensitivity Analysis for Zimbabwe (excess deaths). Sensitivity analysis, for Zimbabwe, exploring the effect of [file pmed.1004334.s002.docx]
