# Supplementary material for: Estimating the impact of alternative programmatic cotrimoxazole strategies on mortality among children born to mothers with HIV: A modelling study
Source: PLoS Med. 2024 Feb 20;21(2):e1004334. doi: 10.1371/journal.pmed.1004334 (PMC10914273; doi:10.1371/journal.pmed.1004334)
Supplement: Attachment — Submitted filename: Review Plos Medicine.docx [file pmed.1004334.s003.docx]

The manuscript reports the impact of different cotrimoxazole strategies on mortality among children born to HIV-positive mothers. This work may help inform WHO recommendations on cotrimoxazole prophylaxis given some concerns on its necessity among HIV-exposed children given concerns on antibiotic resistance.

The manuscript is well written and informative: I only have minor comments regarding the methods:

Model structure

In my opinion, the author should clearly state what they consider as “early initiation diagnosis (EID)” before detailing the model in order to clarify it.

Two model structures are provided: Figure 1 and Supplementary table 1. In my opinion, providing two model structures with the same information may be misleading and authors may consider retaining only one or clearly explaining the differences between both. Regarding the model structure in Supplementary Material, the document states that infants are categorized according to whether they have acquired perinatal HIV and whether they undergo early infant diagnosis. If I understand well, perinatal HIV is defined as acquiring HIV by 6 weeks, but the model classifies the children as born with HIV or not, which in my opinion is different.

Data sources and parameters

Data on subsequent testing after EID and engagement of children with HIV services: in my opinion it would be important to support the assumptions with literature. For Mozambique, I suggest to read the “*Inquérito de Indicadores de Imunização, Malária e HIV/SIDA*” (https://www.misau.gov.mz/index.php/inqueritos-de-saude?download=566:relatorio-final-imasida). Despite it dates from 2015, I think it might provide useful information to inform the assumptions.
