# Supplementary material for: Estimating the impact of alternative programmatic cotrimoxazole strategies on mortality among children born to mothers with HIV: A modelling study
Source: PLoS Med. 2024 Feb 20;21(2):e1004334. doi: 10.1371/journal.pmed.1004334 (PMC10914273; doi:10.1371/journal.pmed.1004334)
Supplement: Attachment — Submitted filename: CTX Modelling PLoS Medicine REVISION RESPONSE.docx [file pmed.1004334.s004.docx]

Dear Dr Gaynor-Brook and Editorial Office,

We thank you and the reviewers for the thoughtful comments and feedback which are instrumental to improving the quality of this manuscript. Please find enclosed our manuscript entitled: “Estimating the Impact of Alternative Programmatic Cotrimoxazole Strategies on Mortality Among Children Born to Mothers with HIV: A Modelling Study”. We have revised the manuscript and we describe the revisions to each of the comments below.

We hope that the revised manuscript is suitable for publication. We look forward to hearing from you.

Sincerely,

Dr Shrey Mathur MD

Requests from the editors:

General comments:
Throughout the paper, please adapt reference call-outs to the following style: "... HIV-free [4,5]." (noting the absence of spaces within the square brackets).
Please revise ‘impact’ to ‘potential impact’ or similar when referring to your own results
Throughout the main text, please refer to ‘predicted mortality’ when referring to your own results

Thank you. We have made these changes throughout.

Data availability:
PLOS Medicine requires that the de-identified data underlying the specific results in a published article be made available, without restrictions on access, in a public repository or as Supporting Information at the time of article publication, provided it is legal and ethical to do so. If the data are freely or publicly available, please note this and state the location of the data (include the DOI or accession number); please note that a study author cannot be responsible for permitting access to the data or be the contact person for the data (I note that Andrew Prendergast approved my access request on OSF).

We apologise for these issues around access and their impact on the review process. We thought the Open Science Framework post had been made open access, but we had the wrong approval settings set up, which has now been fixed. We have therefore made the dataset fully public so that it can be accessed without restriction to access. We have also removed non-authors from named project access to preserve their anonymous contribution to the review process going forward.

Title: Please revise your title according to PLOS Medicine's style. We suggest “Estimating the Impact of Alternative Programmatic Cotrimoxazole Strategies on Mortality Among Children Born to Mothers with HIV: A Modelling Study” or similar

Thank you. We have updated the title, as suggested, to “Estimating the Impact of Alternative Programmatic Cotrimoxazole Strategies on Mortality Among Children Born to Mothers with HIV: A Modelling Study”

Abstract:
Line 48 - please revise to ‘potential impact’

Thank you, we have revised to “potential impact” [Page 2, Line 48].

In the last sentence of the Abstract Methods and Findings section, please describe 2-3 of the main limitations of the study's methodology.

We have added this section, which now reads: “The study is limited by uncertain estimates of cotrimoxazole coverage in programmatic settings; an inability to model increases in mortality arising from antimicrobial resistance due to limited surveillance data in sub-Saharan Africa; and lack of a formal health economic analysis." [Page 2, Lines 67-70]

Abstract Conclusions: Please address the implications of your study, being careful to avoid assertions of primacy.

Thank you. We have changed our Conclusion [Page 3, Lines 74-77] to: “Changing current guidelines from universal cotrimoxazole provision for children who are HIV-exposed is predicted to increase mortality across the four modelled high-burden countries, particularly where test-to-treat gaps exist and vertical transmission remains high. These findings can help inform policymaker deliberations on cotrimoxazole strategies in different settings.”

Author Summary:
At this stage, we ask that you include a short, non-technical Author Summary of your research to make findings accessible to a wide audience that includes both scientists and non-scientists. The Author Summary should immediately follow the Abstract in your revised manuscript. This text is subject to editorial change and should be distinct from the scientific abstract. Please see our author guidelines for more information: [https://eur01.safelinks.protection.outlook.com/?url=https%3A%2F%2Fjournals.plos.org%2Fplosmedicine%2Fs%2Frevising-your-manuscript%23loc-author-summary&data=05%7C01%7C%7Cd43aab8dc5c64f5c76fe08dba3cf90e3%7C569df091b01340e386eebd9cb9e25814%7C0%7C0%7C638283885368427952%7CUnknown%7CTWFpbGZsb3d8eyJWIjoiMC4wLjAwMDAiLCJQIjoiV2luMzIiLCJBTiI6Ik1haWwiLCJXVCI6Mn0%3D%7C3000%7C%7C%7C&sdata=qdHTBO01sqphbhotfM%2Fbv5uXakoiMj6XAz4ywdkx2cU%3D&reserved=0](https://journals.plos.org/plosmedicine/s/revising-your-manuscript#loc-author-summary)
In the final bullet point of ‘What Do These Findings Mean?’, please describe the main limitations of the study in non-technical  language.

We have added this section as requested to the revised manuscript.

Introduction:
If there has been a systematic review of the evidence related to your study (or you have conducted one), please refer to and reference that review and indicate whether it supports the need for your study.

Thank you for this suggestion. We have added the following to the Introduction [Page 7, Lines 158-159]: “A recent systematic review highlighted the lack of mortality benefit from cotrimoxazole for children who are HEU [14].”

Methods:
Did your study have a prospective protocol or analysis plan? If so, please include the relevant prospectively written document with your revised manuscript as a Supporting Information file to be published alongside your study, and cite it in the Methods section. Changes in the analysis-- including those made in response to peer review comments-- should be identified as such in the Methods section of the paper, with rationale.

The study did not have a prospective protocol.

We suggest that TRIPOD may be an appropriate checklist for your study design; please include a completed checklist as a supplementary file. Please add the following statement, or similar, to the Methods: "This study is reported as per the Transparent reporting of a multivariable prediction model for individual prognosis or diagnosis (TRIPOD) guideline (S1 Checklist)." The TRIPOD guideline can be found here: [https://eur01.safelinks.protection.outlook.com/?url=https%3A%2F%2Fwww.equator-network.org%2Freporting-guidelines%2Ftripod-statement%2F&data=05%7C01%7C%7Cd43aab8dc5c64f5c76fe08dba3cf90e3%7C569df091b01340e386eebd9cb9e25814%7C0%7C0%7C638283885368427952%7CUnknown%7CTWFpbGZsb3d8eyJWIjoiMC4wLjAwMDAiLCJQIjoiV2luMzIiLCJBTiI6Ik1haWwiLCJXVCI6Mn0%3D%7C3000%7C%7C%7C&sdata=UA47t%2B4K7zzicqO9LYbN4xpuOdPQou9j%2BnRfE8ALafU%3D&reserved=0](https://www.equator-network.org/reporting-guidelines/tripod-statement/)  When completing the checklist, please use section and paragraph numbers, rather than page numbers which will likely no longer correspond to the appropriate sections after copy-editing.

Thank you. We have completed the TRIPOD Checklist with section and paragraph numbers for inclusion in the Supplement.

Using guidance from Geoffrey P Garnett, Simon Cousens, Timothy B Hallett, Richard Steketee, Neff Walker. Mathematical models in the evaluation of health programmes. (2011) Lancet DOI:10.1016/S0140-6736(10)61505-X;                  
Please provide a diagram that shows the model structure, including how the disease natural history is represented and how the putative intervention (cotrimoxazole) could affect the system.

Thank you. Model structure is shown in Fig 1, which we have updated. Panel A shows scenarios of HIV acquisition, model compartments, and mortality probabilities due to natural history and interventions (incl. antiretroviral therapy and cotrimoxazole) as denoted by superscripts. Panel B shows testing time points and periods of cotrimoxazole provision under different Strategies.

Please provide a complete list of model parameters and important caveats about the use of these values noted.

Model parameters are listed in Supplementary Table S1 Model Assumptions and Data Sources. We have included Caveats.

Please provide a clear statement about how the model was fitted to the data (may include goodness-of-fit measure, the numerical algorithm used, which parameter varied, constraints imposed on parameter values, and starting conditions)

Our modelling approach creates a deterministic compartmental model and is formed using assumptions. It is not fitted to data so goodness of fit measurements etc. are not applicable. We have however explored the robustness of inferences to our assumptions using sensitivity analysis and outlined limitations of the model within the manuscript.

For uncertainty analyses, please state the sources of uncertainties quantified and not quantified [can include parameter, data, and model structure].

In Supplementary Table S1 Model Assumptions and Data Sources, we have identified the data sources for each parameter. We have explored the robustness of inferences to our assumptions using sensitivity analysis and discussed our findings within the manuscript in the Methods section.

Please discuss the scientific rationale for this choice of model structure and identify points where this choice could influence conclusions drawn. Please also describe the strength of the scientific basis underlying the key model assumptions.

Our aim was to explore the impact of alternative programmatic cotrimoxazole strategies on mortality among children born to mothers with HIV. Due to ethics and the limitation of available data reflecting the alternative programmatic strategies that we wish to explore, we chose a deterministic compartmental modelling approach. The framework seen in Figure 1 was constructed from experts working in the contextual field and we have discussed the limitation and assumption in detail within the manuscript, and included sensitivity analyses to explore the robustness of our conclusions.

Results:
Line 236 - please clarify that sensitivity analyses for Mozambique are shown in Fig 3

We have updated the text – Page 11, Line 298.

Throughout the results section, please refer to ‘predicted mortality’

Thank you, we have amended this.

Please provide tables/figures for all of the sensitivity analyses presented in the main text (these can be included in the supplementary information)

Thank you. In addition to presenting sensitivity analysis for Mozambique in the main text, we have included tables and figures for all sensitivity analyses in the supplement.

Line 252 - please refer to specific supplementary figures for each of these countries. NB) these supplementary figures have not been included in this submission.

Thank you; we have included specific supplementary figures for each of the countries.

Discussion:
Please present and organize the Discussion as follows: a short, clear summary of the article's findings; what the study adds to existing research and where and why the results may differ from previous research; strengths and limitations of the study; implications and next steps for research, clinical practice, and/or public policy; one-paragraph conclusion.

Thank you for this suggestion. We have restructured the discussion as advised.

Throughout the discussion, please refer to ‘predicted mortality’ when referring to your own results

Thank you, we have updated this.

Figures:
Please consider avoiding the use of red and green in order to make your figure more accessible to those with colour blindness.

We have changed figure colours to improve accessibility for those with colour blindness.

Please define all abbreviations used in the figure legend of each figure.

Thank you; we have defined all abbreviations in the figure legends.

Tables:
Please define all abbreviations used in the table legend of each table (including supplementary tables).

Thank you; we have defined all abbreviations for figures and tables, including those in the supplement.

References:
Please ensure that journal name abbreviations match those found in the National Center for Biotechnology Information (NCBI) databases ([https://eur01.safelinks.protection.outlook.com/?url=http%3A%2F%2Fwww.ncbi.nlm.nih.gov%2Fnlmcatalog%2Fjournals&data=05%7C01%7C%7Cd43aab8dc5c64f5c76fe08dba3cf90e3%7C569df091b01340e386eebd9cb9e25814%7C0%7C0%7C638283885368427952%7CUnknown%7CTWFpbGZsb3d8eyJWIjoiMC4wLjAwMDAiLCJQIjoiV2luMzIiLCJBTiI6Ik1haWwiLCJXVCI6Mn0%3D%7C3000%7C%7C%7C&sdata=C91UIpfFmMJ%2BoL6BEvyKCxiaSA0IDJfYYeRxCKxiPbo%3D&reserved=0](http://www.ncbi.nlm.nih.gov/nlmcatalog/journals)), and are appropriately formatted and capitalised.
Please also see [https://eur01.safelinks.protection.outlook.com/?url=https%3A%2F%2Fjournals.plos.org%2Fplosmedicine%2Fs%2Fsubmission-guidelines%23loc-references&data=05%7C01%7C%7Cd43aab8dc5c64f5c76fe08dba3cf90e3%7C569df091b01340e386eebd9cb9e25814%7C0%7C0%7C638283885368427952%7CUnknown%7CTWFpbGZsb3d8eyJWIjoiMC4wLjAwMDAiLCJQIjoiV2luMzIiLCJBTiI6Ik1haWwiLCJXVCI6Mn0%3D%7C3000%7C%7C%7C&sdata=rZ7oRa7BqZwpuvD2MDz%2FJBFEYcBBTuQ2CEytXCLrgwI%3D&reserved=0](https://journals.plos.org/plosmedicine/s/submission-guidelines#loc-references) for further details on reference formatting.
Where website addresses are cited, please specify the date of access.

Thank you, we have updated the references and added date of access for website addresses.

Comments from the reviewers:

**Reviewer #1:** Mathur and colleagues describe in this manuscript deterministic compartmental models developed by the authors relying upon up-to-date, publicly available estimates including of HIV early infant HIV testing (EIT), prevalence of infant HIV acquisition, and antiretroviral treatment (ART) uptake to evaluate mortality  between 6- and 24-months of life among HIV-exposed children comparing a strategy of universal cotrimoxazole use for all children to other strategies with lower coverage, as cotrimoxazole has not bene shown to have any survival benefit when used in children who are HIV-exposed uninfected, but has been shown to improve survival in children living with HIV not yet on treatment.  Models were applied to four high burden HIV-countries yet to achieve 95-95-95 HIV program targets or Eliminating Mother-to-Child-Transmission goals, specifically Cote d'Ivoire, Mozambique, Uganda, and Zimbabwe. Country-specific data was supplemented with data extracted from peer-reviewed publications of clinical trials, observational studies, and other epidemiological data in eras both before and after universal ART was recommended at time of HIV diagnoses.  The paper is well-written with excellent tables and figures. Considerable work has been devoted to testing multiple strategies. The authors conclude changing the current recommendations of universal cotrimoxazole provision for all HIV-exposed infants would increase mortality to varying degrees depending on vertical transmission rates and test-to-treatment cascade strategies would increase mortality to varying degrees.  While this is an important manuscript that will be of interest to PLOS Medicine readers, there are two issues raised by the final recommendation of the modeling exercise that need to be addressed by the authors. Major and minor revision recommendations are presented below.                                                                                                                                                                                                                                                                                                                                                                Major Revision Recommendations:
The authors' final conclusions advocate for universal cotrimoxazole use by HIV-exposed infants until infection is excluded and vertical transmission risk has ended. Conflictingly, the authors make mention in the discussion that among countries achieving 95-95-95 testing and treatment targets and Elimination of Mother-to-Child goals, cotrimoxazole prophylaxis may not be required.  Interestingly, in at least one of the two studies cited for the lack of survival benefit of cotrimoxazole use among infants and children HIV-exposed uninfected, the Mpepu randomized controlled trial was conducted in a setting where the 95-95-95 targets have been achieved and infant HIV acquisition is currently estimated to be approximately 1%.  This presents an argument for a hybrid policy, as the absence of using a country such as Botswana in the modeling exercise may be influencing an incorrect policy recommendation about universal use of cotrimoxazole.

Thank you for this helpful comment. There has been rapid progress in response to the HIV epidemic. Botswana is the first high-burden setting to reach these targets and its laudable progress may not be reflective of the state of the epidemic in other settings. Furthermore, Botswana continues to have high prevalence of HIV among women of childbearing age and 74% of infants receiving Early Infant Diagnosis (2022, <https://www.unaids.org/en/regionscountries/countries/botswana>, Accessed 2023.09.03).

In the sensitivity analysis, we have interrogated the effect of reduced transmission perinatal and postnatal transmission rates and have noted that though effects are attenuated, mortality is predicted to increase with alternative cotrimoxazole strategies. Deaths may be driven here by children who are missed by testing and gaps in the test-to-treatment cascade.

A hybrid policy contingent on transmission and testing rates based on targets may be tenable in a few settings in the future. However, even settings with exceptional progress toward targets, such as Botswana, continue to have gaps in testing for which the current CTX provides a safety net.

We have added the following to the Discussion [Page 14, Lines 415-423], “As demonstrated in the sensitivity analysis for each strategy, lower vertical transmission rates and improved testing and treatment uptake attenuate the predicted increase in mortality. This demonstrates the importance of considering how vertical transmission should inform cotrimoxazole policy: in the context of high ongoing transmission, the current universal cotrimoxazole prophylaxis approach is predicted to lead to substantially fewer deaths than alternative strategies, while settings with low transmission rates may derive less benefit from universal cotrimoxazole prophylaxis. Therefore, hybrid cotrimoxazole policies may be a consideration noting we did not model countries achieving HIV elimination targets or with strong test-to-treat cascades.”

The model assumes that 100% of infants who are HIV-exposed and for whom HIV infection status has not been finalized or vertical transmission risk continues to exist will be prescribed cotrimoxazole and parents or caregivers will have 100% adherence to dosing. While the authors acknowledged that they lacked a data source to estimate the number of children most likely to acquire HIV who are not engaged in care, this is a major flaw in the model that leads to questionable justification for continued universal cotrimoxazole use.  If there is an issue with timely HIV testing of infants at risk for HIV, or timely ART initiation for those who found to be infected, monthly or quarterly encounters with the health care system for refills of cotrimoxazole are highly unlikely.  If they are occurring, they could be better spent employing HIV testing or initiation of treatment, as applicable.  It would be important in the Discussion section to call for accurate accounting of cotrimoxazole
use in HIV-exposed infants to determine the proportion who actually receive this treatment and are subsequently found to be living with HIV, with a further understanding of the hindrances that preclude timely diagnoses and treatment initiation.  The elephant in the room is engagement in the care cascade.  To justify a policy that, on paper appears to save lives, but exchanges one medication regimen, namely ART, for another, cotrimoxazole, promulgates continued weaknesses in the treatment and care cascade and may only save lives on paper.

Thank you for this comment. We agree that gaps in the test-to-treat cascade increase the risk of mortality. We also agree that further research is needed to define cotrimoxazole uptake as we were unable to find data to incorporate into the model. The Reviewer is correct that the *base* assumption for the purposes of the model is 100% cotrimoxazole uptake. However, in the sensitivity analysis, we have explored much lower levels of cotrimoxazole uptake (as low as 30%) which may be more reflective of many settings. At these low rates of uptake of cotrimoxazole, it is possible for readers to ascertain the estimated number of deaths that are modelled, but we recognise that this is one factor within many variables that need to be considered when amending guidelines.

We have added the following sentence to the Discussion [Page 14, Lines 422-428], “Further research is required to better understand cotrimoxazole coverage for children who are HIV-exposed; to determine the proportion who are subsequently found to be living with HIV; to better understand community values and preferences; and to identify barriers to engagement with the test-to-treat cascade. Further cost-effectiveness analysis informed by national programme cost data is required to better understand cost per death averted and economic impact of each strategy.”

Minor Revision Recommendations:
Please use person first language throughout the manuscript. For example, at line 45 reference is made for "cotrimoxazole prophylaxis for HIV-exposed infants".  Please revised this to read "cotrimoxazole prophylaxis for infants who are HIV-exposed".

In the Abstract, we have changed “HIV-exposed infants“ to “children who are HIV-exposed” [Page 2, Lines 45-46] and “HIV-exposed uninfected children” to “children who are HIV-exposed uninfected” [Page 2, Lines 47-48].

Please review references, as some have "abbreviated" authorship.  (eg references 1, 2, 15 and 20).

Thank you for spotting this. We have updated the references.

At lines 349-350, reference is made to "EMTCT Path to Elimination goals".  However, since the "E" in EMTCT stand for Elimination, this does not make sense. It might be clearer to take out the word "Elimination" in this phrase.

Thank you for this suggestion. We have rephrased, to ‘Path to Elimination of MTCT’ [Page 13, Lines 382-383].

**Reviewer #2:** This paper presents the mortality effects of providing cotrimoxazole for periods shorter than the WHO guidelines. The conclusion is obvious, shorter periods of cotrim results in more deaths, but the paper is useful for showing the magnitude of the effect.

1. In my opinion the paper is not really an evaluation of the WHO guidelines because it doe not consider any costs or negative effects. It would be relatively easy to show the additional costs of longer periods of cotrim and the cost per death averted. Since cotrim is inexpensive I assume it is very cost-effective. The authors do not include increased antimicrobial resistance because they are unsure of the effect size. But they did extensive sensitivity analysis of the model parameters. Why not include sensitivity around assumptions about resistance? It would make the paper much stronger.

Thank you for this helpful comment which highlights some limitations of the model and the important consideration of antimicrobial resistance (AMR), cost and guideline development.

Our major challenge for including an effect of AMR is data availability to inform effect sizes and reversibility of any resistance that may directly arise due to different durations of cotrimoxazole use under our six scenarios. Surveillance data regarding resistance in LMICs is limited. The most valuable resource is the recent Global Burden of AMR (<https://doi.org/10.1016/S0140-6736(21)02724-0>), which modelled global deaths attributable to bacterial antimicrobial resistance by pathogen–drug combination for the sub-Saharan Africa super-region. They reported pathogen-specific deaths (rate per 100 000) attributable to AMR from cotrimoxazole in sub-Saharan Africa in the post-neonatal period as *Proteus spp.* 0.0 (0.0-0-1), *Enterobacter spp.* 0.3 (0.0-0.7), *Escherichia coli* 3.2 (2.1-4.8), *Klebsiella pneumoniae* 3.5 (1.7-5.5), *Staphylococcus aureus* 4.2 (2.2-6.5), and *Streptococcus pneumoniae* 15.7 (2.3-31.8). Collectively, these estimates are much lower than even the most conservative mortality increases found in our modelling. Incorporating them meaningfully into our model, however, provides several challenges: first, GBD provides modelled data for a limited range of pathogens; second, their data do not specifically allow us to estimate what proportion of these deaths would be *averted* with a change in cotrimoxazole policy for HIV-exposed infants; third, it remains unclear whether cotrimoxazole resistance is reversible to a clinically relevant level with decline in use (<https://doi.org/10.1016/j.jgar.2017.07.013>); and fourth, our model reflects mortality in the first year following policy change whereas modelling deaths due to AMR would involve forward predictions.

We cannot therefore find a way to meaningfully incorporate these estimates into our model. However, the Reviewer’s point is well taken, and we have paid more attention to the important issue of AMR in the revision. We have added the following sentence to the Discussion [Page 13, Lines 392-403]: This expanded section now reads: “There is evidence that cotrimoxazole use in HEU infants leads to antimicrobial resistance, including one small study showing cross-class resistance to amoxicillin, but no evidence of major microbiome dysbiosis from available data [23]. Although there are estimates of deaths attributable to resistance for specific antibiotics in several global regions [24], it is difficult to model what proportion of these deaths would be averted in each of our scenarios, particularly as it remains unclear whether cotrimoxazole resistance is reversible to a clinically relevant level with decline in use [25]. Estimates of pathogen-specific deaths attributable to AMR from cotrimoxazole in sub-Saharan Africa in the post-neonatal period range from 0.0 (0.0-0.1) per 100 000 for *Proteus spp.* to 15.07 (2.3-31.8) per 100 000 for *Streptococcus pneumoniae*, but even collectively these estimates are much lower than the excess deaths modelled in our programmatic scenarios from restricted cotrimoxazole use. Responsive country-level surveillance is required to evaluate local resistance data in the context of evolving HIV epidemics.”

Cost of cotrimoxazole would be informed by costs of the drug (based on procurement, dose regimen, formulation), and healthcare costs (eg. outpatient visits), and programme costs. We did not have access to national programme cost data to inform cost-effectiveness analysis, but we agree this is a priority research question and have added the following to the Discussion [Page 14, Lines 427-428], “Further cost-effectiveness analysis informed by national programme cost data is required to better understand cost per death averted and economic impact of each strategy.”

We agree that an evaluation of guidelines would have further considerations, including community values and preferences. We have added to the Discussion [Page 14, Lines 408-409], “Results from this model may be considered as part of the breadth of evidence which help inform policymaker decisions.”

2. The paper states that the model is freely available and provide a website. I tried to download the model but have been unsuccessful so far. I went to the OSF website using the link provided in the paper, but had to first create an account with OSF. Once I did that I received a message that I had to request permission to review the file. I requested permission. The next day I was informed that permission had been granted. When I went to the site I just saw a folder that had been set up for collaboration. There was no Excel file in that folder. So I still have not seen the model. Other readers may be frustrated with this process. Since I had to request permission there was no hope of a confidential review.

We have now made the dataset publicly available and apologise for the difficultly in accessing the dataset, which was due to an incorrect permissions setting on OSF when we first created the site.

**Reviewer #3:** The study appears to be highly relevant and valuable in informing the ongoing debate regarding the revision of the current WHO policy on administering cotrimoxazole to all HIV-exposed infants during breastfeeding. I believe that the research findings will serve as a credible and significant piece of evidence for understanding the disease and its treatment. Here are several suggested comments that the authors could address to enhance the manuscript:

1.      The manuscript requires revisions to enhance clarity and readability. The authors should clarify certain sections to ensure their intended meaning is clear to the reader. Additionally, there are ambiguous statements that need to be revised for better comprehension. Coherence should be improved to ensure a logical flow of ideas between paragraphs. By addressing these specific areas for improvement, the authors can significantly enhance the overall quality of the manuscript, making it more accessible to readers. I have provided specific examples below:

a)      Until the elimination of vertical transmission is achieved, therefore, children born to mothers with HIV require programmatic strategies to reduce morbidity and mortality.

Thank you for these suggestions. Rephrased for clarity to “Given ongoing high transmission rates, limited PMTCT uptake, and rapid progression in undiagnosed children, programmatic strategies are needed to reduce morbidity and mortality among children born to mothers with HIV, until elimination of vertical transmission is achieved.” [Page 6, Lines 133-136]

b)      There is therefore a critical need to identify and distinguish children with HIV from children who are HEU across different periods of transmission, given the distinct cotrimoxazole strategy required in each group.

Rephrased to “Given the distinct cotrimoxazole strategy required in each group, there is a critical need to identify and distinguish children with HIV from children who are HEU across different periods of transmission.” [Page 6, Lines 146-148].

c)      Even where infants are tested, challenges remain across the testing-to-treatment cascade, with delay in the return of results, lack of integration with child health services, loss to follow-up, and delayed ART initiation.

We have split this into two sentences. Introduction, Page 6, Lines 151-154: “Even where infants are tested, challenges remain across the testing-to-treatment cascade. These include lengthy turnaround times for results, lack of integration with child health services, loss to follow-up, and delayed ART initiation [12,13].”

d)      The model starts at age 6 weeks when early infant diagnosis and cotrimoxazole initiation are recommended and estimates mortality to 24 months of age

We have rephrased to: “The model estimates mortality from 6 weeks of age, when cotrimoxazole initiation is recommended, to 24 months of age.” [Page 7, Lines 179-180]

e)      Sensitivity analysis (results) paragraph one: "Reducing perinatal and postnatal vertical transmission rates towards elimination targets and beyond decreased the effect of cotrimoxazole on mortality until it reached zero effect at 0% transmission."

Rephrased to: “To explore the effect of reaching and exceeding MTCT elimination targets, we reduced rates for both perinatal and postnatal vertical transmission to zero.” [Page 11, Lines 305-306]

f)      Once HIV was diagnosed (by 9 months or EoB), 76% mortality reduction from ART plus 43% mortality reduction from cotrimoxazole was applied, for the 77% of children with HIV who start ART

Rephrased to “With the same rationale as in the first scenario, for 77% of children diagnosed with HIV (by 9 month or EoB testing), we applied mortality reduction of 76% from ART and 43% from cotrimoxazole.” [Page 9, Lines 242-244]

2.      The authors referenced "expert opinion" as a data source in Supplementary Table 1. To improve clarity, it is recommended to provide additional details in the method section regarding the nature of the experts used and how "expert opinion" was utilised to extract data. Specifically, explain the qualifications or expertise of the experts involved and describe the process by which their opinions were obtained and incorporated into the analysis. Additionally, within the manuscript, it would be beneficial to clarify the assumptions ("Since data on subsequent testing are not available, we assumed that 80% of children undergoing EID would have a 9-month test, and 50% would have an EoB test, as these children would likely be engaged in the test-to-treatment cascade. For children not undergoing 162 EID at 6 weeks, we assumed the probability of subsequent testing was much lower (10% for 9-month 163 test, 30% for EoB), as these children were likely less engaged with services.") that were made for testing if they are based on expert opinion. This will help readers understand the methodology and the basis for the analysis, ensuring transparency and reproducibility.

Thank you for this comment. The authorship brings multidisciplinary expertise in paediatric HIV (AJP, CE, DMG, CW, MP), epidemiology (MS, DMG), clinical trials (DMG, AJP, MP, MS) and policy (SM, MP). Where data were not available, we estimated parameters based on our collective knowledge and applied sensitivity analyses to interrogate these assumptions. We have added comments and caveats to Supplementary Table S1 Model Assumptions and Data Sources.

3.      The authors state that in all strategies, they assume that infants testing positive for HIV will initiate cotrimoxazole treatment. However, it would be beneficial for the manuscript to explicitly mention the probability of an HIV test yielding a positive result and provide clarification regarding the specific time points at which testing was assigned in the model. This additional information will enhance the transparency of the study and enable readers to better understand the assumptions made regarding HIV test outcomes and the timing of testing within the modeling.

Thank you for this helpful suggestion. Regarding testing time points, we have added the following text to Methods, Model Structure [Page 7, Lines 180-181]: “Testing timepoints were assigned at 6 weeks (defined as early infant diagnosis, EID), at 9 months and at 18 months (defined as end of breastfeeding, EoB).”

Please see comment 4 below regarding HIV test outcomes.

4.      If the following details mentioned inside the manuscript are linked with comment 3 please revise the manuscript accordingly. "To determine the probability of a child testing HIV-positive by age 6 weeks, perinatal transmission rates were obtained from country-specific UNAIDS estimates from the most recent year with available data for all countries, including six-week transmission rates (MOZ 6%, ZWE 152 5%, CIV 4%, and UGA 3%) (2). For postnatal transmission, we calculated the cumulative probability of acquiring HIV by age 9 months (MOZ 3.4%, ZWE 1.7%, CIV 1.7%, UGA 1.3%), and by the end of breastfeeding (MOZ 4.1%, ZWE 2.0%, CIV 2.1%, UGA 1.6%), based on derived weekly transmission rates from country-specific final vertical transmission rates and breastfeeding duration, making the assumption that postnatal transmission rates were consistent over time."

Thank you. We have added the following sentence to immediately precede the text above in Methods, Data Sources and Parameters [Page 8, Lines 210-213]. “The probability of an HIV test yielding a positive result was based on age-specific transmission rates, obtained from country-specific UNAIDS estimates from the most recent year with available data for all countries, including six-week transmission rates (MOZ 6%, ZWE 5%, CIV 4%, and UGA 3%) [2].”

5.      The authors mentioned in the manuscript that globally, 62% of infants currently receive the recommended virological test within two months of birth, and as a result, 40% of children with HIV remain undiagnosed. It would be helpful if the authors could clarify whether these specific values were utilized within the model being discussed in the study. Providing this clarification will enable readers to better understand the extent to which these statistics were incorporated into the modeling process.

Thank you for this comment. We added the global statistics to inform the reader about the wider context and how impactful and important the topic is. We did not use the global statistics within our model, instead we used country-specific data for the four settings for testing rates to improve our estimations. We list all estimates used in Supplementary Table S1 but have added wording on Lines 176-177 to clarify this.

6.      Please clarify within the manuscript whether the model explicitly incorporates the following strategies: providing antiretroviral therapy (ART) for undiagnosed cases and cotrimoxazole for cases testing positive for HIV, as well as whether it considers the progression of the disease among children with HIV. This clarification is important for understanding the interventions considered in the study and their potential impact on disease progression and treatment outcomes.

Thank you for this comment. We have amended the text in Methods, Model Structure [Page 7, Lines 184-189] to clarify these issues, adding: “Cotrimoxazole was provided based on programmatic strategy (Fig 1B). If the child was diagnosed with HIV, cotrimoxazole was started in conjunction with ART. ART was initiated only after a positive HIV test and was not initiated after negative or missed tests (i.e., for undiagnosed cases). Disease progression was incorporated through survival probabilities for children with HIV and children who are HIV-exposed uninfected, extracted from trial data and natural history studies [3,15].”

7.      Please clarify within the manuscript whether the model explicitly incorporates the following strategies among children with HIV:

a)      Providing antiretroviral therapy (ART) for undiagnosed cases and cotrimoxazole for cases testing positive for HIV

Thank you. We have amended the text in Methods, Model Structure [Page 7, Lines 184-187] to clarify these issues, adding: “Cotrimoxazole was provided based on programmatic strategy (Fig 1B). If the child was diagnosed with HIV, cotrimoxazole was started in conjunction with ART. ART was initiated only after a positive HIV test and was not initiated after negative or missed tests (i.e., for undiagnosed cases).”

b)      Disease progression among children with HIV into the mode

We have added to Methods, Model Structure [Page 7, Lines 187-189], “Disease progression was incorporated through survival probabilities for children with HIV and children who are HIV-exposed uninfected, extracted from trial data and natural history studies [3,15].”

8.      Could the author kindly include the confidence interval (CI), if possible, for the risk ratio of strategies in comparison to the current strategy?

Thank you for this comment. The method we have used is deterministic and therefore does not have uncertainty incorporated, thus confidence intervals are not feasible for this type of method. We have however run a number of sensitivity analyses to explore the robustness (uncertainty) of our inferences (including the risk ratio) to varying assumptions within the model.

9.      Could the authors please provide a summary of the main findings? This summary should be provided as a paragraph at the beginning of the discussion section.

Thank you for this suggestion. We have restructured the Discussion and provided a summary in the first paragraph.

10.     Could the authors please provide a clear and detailed discussion of the limitations of the research? Additionally, it would be valuable to explore the need for further research, such as conducting a cost-effectiveness analysis and examining the potential impact on antibiotic resistance for the proposed strategies.

Thank you. This section has been revised and now provides a discussion of the study limitations.

**Reviewer #4:** The manuscript reports the impact of different cotrimoxazole strategies on mortality among children born to HIV-positive mothers. This work may help inform WHO recommendations on cotrimoxazole prophylaxis given some concerns on its necessity among HIV-exposed children given concerns on antibiotic resistance.
The manuscript is well written and informative: I only have minor comments regarding the methods:
Model structure
In my opinion, the author should clearly state what they consider as "early initiation diagnosis (EID)" before detailing the model in order to clarify it.

We have added the follow text to Methods, Model Structure [Page 7, Lines 180-181]: “Testing timepoints were assigned at 6 weeks (defined as early infant diagnosis, EID), at 9 months and at 18 months (defined as end of breastfeeding, EoB).”

Two model structures are provided: Figure 1 and Supplementary table 1. In my opinion, providing two model structures may be misleading and authors may consider clearly explaining the differences between both. Regarding the model structure in Supplementary Material, the document states that infants are categorized according to whether they have acquired perinatal HIV and whether they undergo early infant diagnosis. If I understand well, perinatal HIV is defined as acquiring HIV by 6 weeks, but the model classifies the children as born with HIV or not, which in my opinion is different.

Thank you. We agree that two model structures could be confusing, so in the revised manuscript we have incorporated elements of the Supplementary Figure into Fig 1. There is now therefore only one model shown (Fig 1).

Data sources and parameters
Data on subsequent testing after EID and engagement of children with HIV services: in my opinion it would be important to support the assumptions with literature. For Mozambique, I suggest to read the "Inquérito de Indicadores de Imunização, Malária e HIV/SIDA" (<https://eur01.safelinks.protection.outlook.com/?url=https%3A%2F%2Fwww.misau.gov.mz%2Findex.php%2Finqueritos-de-saude%3Fdownload%3D566%3Arelatorio-final-imasida&data=05%7C01%7C%7Cd43aab8dc5c64f5c76fe08dba3cf90e3%7C569df091b01340e386eebd9cb9e25814%7C0%7C0%7C638283885368427952%7CUnknown%7CTWFpbGZsb3d8eyJWIjoiMC4wLjAwMDAiLCJQIjoiV2luMzIiLCJBTiI6Ik1haWwiLCJXVCI6Mn0%3D%7C3000%7C%7C%7C&sdata=6ZZQa6lCzhHNe5zWttlZ8KAdGvLMCWdeRaKmKPKN3mo%3D&reserved=0>). Despite it dates from 2015, I think it might provide useful information to inform the assumptions.

Thank you for this helpful suggestion. Unfortunately, due to accessibility of this document, which is in Portuguese, we were unable to use it to inform our assumptions. We have interrogated our assumptions, especially where data were not available, in our sensitivity analysis.
