# Supplementary material for: Estimating the impact of alternative programmatic cotrimoxazole strategies on mortality among children born to mothers with HIV: A modelling study
Source: PLoS Med. 2024 Feb 20;21(2):e1004334. doi: 10.1371/journal.pmed.1004334 (PMC10914273; doi:10.1371/journal.pmed.1004334)
Supplement: Attachment — Submitted filename: CTX Modelling PLoS Medicine REVISION2 RESPONSE.docx [file pmed.1004334.s005.docx]

Dear Dr Gaynor-Brook and Editorial Office,

We thank you and the reviewers for the thoughtful comments and suggestions.

Please find enclosed the second revision of our manuscript entitled: “Estimating the Impact of Alternative Programmatic Cotrimoxazole Strategies on Mortality Among Children Born to Mothers with HIV: A Modelling Study.” We have further revised the manuscript and we describe the revisions to each of the comments below.

We hope that the revised manuscript is suitable for publication. We look forward to hearing from you.

Sincerely,

Dr Shrey Mathur MD

Requests from the editors:
In your revised submission, please ensure that you fully address the comments from Reviewer 1, relating to strengthening the model to take into account cotrimoxazole usage among infants not engaged in care, and consideration of the Mpepu RCT as suggested.

Thank you. We have addressed the reviewer comments below.

Comments from the reviewers:

**Reviewer #1:** Mathur and colleagues have significantly improved their manuscript entitled "The Impact of Alternative Programmatic Cotrimoxazole Strategies on Mortality Among Children Born to Mothers with HIV: A Modelling Study" by addressing opportunities raised by the editorial staff of PLOS Medicine, as well as that of the reviewers.  Again noted is the considerable effort in further customizing the multiple strategies to quantify predicted mortality. There remain opportunities to further strengthen the manuscript, including:

The premise of this modeling exercise is that infants and children living with HIV are not diagnosed and treated proximal to the incident HIV infection. While cotrimoxazole does have mortality benefit among children infected with HIV, all models presume there will be no change in pediatric HIV testing and rapid antiretroviral treatment initiation. In the limitations paragraph of the discussion section it should be pointed out that investment in ensuring that all children HIV-exposed uninfected receive cotrimoxazole until risk of HIV infection has been eliminated may not represent the optimal health investment compared to investing in timely testing and treatment, an alternative known to substantially reduce morbidity and mortality, preserve immune function, and limit seeding of the HIV viral reservoir, that latter two of which cotrimoxazole cannot affect.

Thank you for this comment. We agree that a strong test-to-treat cascade is instrumental to reducing mortality. Our study highlights the role of cotrimoxazole in providing a safety net, given current gaps in test-to-treat cascades in high burden settings, but is not intended to replace timely testing and treatment. We have added the following to the Discussion, Lines 434-436: “Cotrimoxazole is one element of the package of care; countries need to continue to strengthen the test-to-treat cascade, to ensure timely testing and treatment initiation, in order to reduce morbidity and mortality.” We have also added to the Author Summary [Lines 120-122]: “Cotrimoxazole continues to provide important protection to children who acquire HIV and are missed by gaps in the test-to-treatment cascade, but does not replace the importance of timely testing and treatment.”

At lines 222 through 224, while it is appropriate for the purposes of modeling to assume that children who do not test at 6 weeks would have a lower prevalence of testing at 9 months and following cessation of breastfeeding, as the authors appropriately note this group is less likely to be engaged in care, it is imperative that this population also have a much lower use of cotrimoxazole in model.  If they are not engaged in care, they will not be prescribed cotrimoxazole. Reducing the proportion of infants in this group who actually take cotrimoxazole will likely substantially reduce the overall mortality benefit of a policy of universal use of cotrimoxazole in high burden HIV settings.

Thank you for this comment. We agree that children who are not engaged with care are less likely to receive cotrimoxazole. There is limited evidence to quantify the degree of cotrimoxazole use for children living with HIV and especially for those not engaged in care or intermittently engaged in care. Limited engagement with care, including intermittent use of cotrimoxazole, can also apply to children who *did* undergo initial EID testing, given that a high proportion are lost to follow-up. It is difficult to account for each of these situations in the primary model. As such, we have interrogated our baseline assumption of 100% cotrimoxazole uptake in each of the four high burden settings in our sensitivity analyses, reducing cotrimoxazole uptake to as low as 40% (Figs S4.1 for Zimbabwe, S4.7 for Cote d’Ivoire, S4.13 for Mozambique, S4.19 for Uganda). As expected, this reduces the mortality benefits of cotrimoxazole, due to lower coverage, but still shows a greater reduction in deaths under the current cotrimoxazole strategy.

This limitation has been acknowledged in Discussion, Lines 387-389: “We assumed equal uptake of cotrimoxazole regardless of final child HIV status, but it is likely that children most likely to acquire HIV are the least engaged in care and are therefore less likely to receive cotrimoxazole.”

At lines 258 and 259, rationale is used for selection of conservative estimates of mortality benefit with cotrimoxazole use, since the CHAP study included very few infants.  Why wouldn't the authors use the Mpepu study, a more current study were infants HIV-exposed uninfected were randomized to cotrimoxazole or placebo through 15 months of life?  This is particularly important as at lines 372 through 374 it is stated that there is no randomized controlled trial data for cotrimoxazole among breastfed infants. The Mpepu study in Botswana was randomized controlled trial that included breastfed infants.

Thank you for this comment. The findings of the Mpepu study in Botswana and the South African study (references 8 and 9) (i.e. that there is no mortality benefit from cotrimoxazole for children who are HEU) have been incorporated into the model throughout. In the model, based on these studies, children who are HEU have a zero percent mortality reduction applied from receiving cotrimoxazole. By contrast, children with HIV *do* have a mortality reduction applied, based on data from the CHAP trial. To inform baseline mortality estimates for children who are HEU, we used Arikawa et al. (reference 15) which is a pooled analysis of individual data on 19 219 HEU children from 21 PMTCT trials/cohorts undertaken from 1995 to 2015 in Africa and Asia. While the Mpepu study includes children up to age 15 months, the Arikawa analysis includes only children up to 24 months which was the mortality endpoint for our analysis.

At lines 361 through 362, in addition to recognizing to the need to focus on reducing vertical transmission, it would be equally important to highlight, where vertical transmission does occur, timely testing and initiation of antiretroviral treatment.

Thank you for this suggestion. We have added to the Discussion, Lines 367-370: “Where vertical transmission does occur, timely testing and initiation of antiretroviral treatment remains a cornerstone of care. Countries with ongoing high rates of transmission would be most affected by any change in cotrimoxazole strategy.”

Under "Why was this study done?", under the first bullet, how would one ascertain the period of HIV infection risk through breastfeeding has ended without performing testing?  It might be clearer to the reader to state "until the child is no longer at risk for HIV acquisition and documentation of a negative HIV test appropriately timed after the risk has concluded is available".

Thank you for this suggestion, and we acknowledge that this first bullet point was unclear as written. We have now updated this to use the precise language in the WHO guidelines. This now states: “Cotrimoxazole prophylaxis is recommended in World Health Organization (WHO) guidelines for all children born to mothers with HIV until HIV infection has been excluded by an age-appropriate HIV test to establish the final diagnosis after complete cessation of breastfeeding.”

For the statement between lines 91-94, it would be important to use the term "model" in place of "determine" and/or to state "would be predicted to increase mortality" rather than "would increase mortality".

Thank you. We have updated the Author Summary, Lines 94-97: “There is a need to model to what extent alternative cotrimoxazole strategies, which either do not provide universal cotrimoxazole for all HIV-exposed infants, or provide it for a shorter duration, would be predicted to increase mortality in different settings among infants who acquire HIV but are undiagnosed.”

At line 100, can you clarify how the model "incorporates the HIV status of the infant"?  All infants/children in the model start off HIV-exposed uninfected and that same bullet point goes on to explain that perinatal and postnatal transmission rates are employed in the model.

Thank you for this comment. The model applies different baseline mortality estimates depending on the HIV status of the infant – this is elaborated upon in Methods, Scenarios. Baseline mortality estimates were taken for (1) children with HIV (diagnosed), (2) children with HIV (undiagnosed), or (3) children HEU. Age-specific baseline mortality estimates for infants with HIV were based on Newell et al. [3] and age-specific baseline mortality estimates for children HIV-exposed uninfected were based on Arikawa et al. [15].

Please consider changing the text at lines 121 and 122 to read "Policymakers need to weigh the risks and benefits of guidelines for cotrimoxazole prophylaxis among infants HIV-exposed uninfected, recognizing that vertical transmission rates and timely test and treat programming significantly influences predicted mortality outcomes".

Thank you we have updated the Author Summary, Lines 125-128. We understand the reviewer’s point here, but our model does not focus only on infants HIV-exposed uninfected, but also on the population of children with undiagnosed HIV. We agree with the point that lower transmission rates and timely testing and treatment critically influence mortality. We have therefore updated the original text to read: “Policymakers need to weigh the risks and benefits of cotrimoxazole prophylaxis through any change to current recommendations, noting that these differ across settings: where lower vertical transmission rates and improved testing and treatment uptake occurs, the estimated mortality benefits of cotrimoxazole are attenuated.”

The text between lines 164 and 166 indicate that modeling of mortality impact of alternative cotrimoxazole strategies in four high-burden settings was performed to inform potential cotrimoxazole recommendations in different contexts. However, modeling in four high-burden settings really does not afford the ability to inform recommendations in different contexts.  It is more accurate to simply state "Here, we model the mortality impact of alternative cotrimoxazole strategies in four high-burden settings".

Thank you. We have updated Introduction, Lines 169-170: “Here, we model the mortality impact of alternative cotrimoxazole strategies in four high-burden settings.” Building on this point, we have also edited the Abstract Conclusion, Lines 74-78 to: “Changing current guidelines from universal cotrimoxazole provision for children who are HIV-exposed increased predicted mortality across the four modelled high-burden countries, depending on test-to-treat cascade coverage and vertical transmission rates. These findings can help inform policymaker deliberations on cotrimoxazole strategies, recognising that the risks and benefits differ across settings.”

The manuscript has many places where first person language is not presented, including at line 92 (HIV-exposed infants should be infants HIV-exposed), line 173, line 204, line 246, and line 392.

Thank you. We have made changes to ensure first person language at Lines 92, 177, 205, 250, 399, and in a few other places where we spotted errors.

PMTCT is used for the first time at line 115. Please spell it out.

Thank you for spotting this. We have made the changes to Lines 1185-119: “… as countries continue to scale up Prevention of Mother to Child Transmission (PMTCT) and EID coverage.”

Lines 180 and 181 do not need "Early Infant Diagnosis" as the abbreviation has already been used previously.

Thank you. We have made this change to Line 184.

At line 87, the "now" is not necessary.

We have removed the word now.

**Reviewer #2:** The authors have done a good job of replying to all my questions and concerns. I have no further concerns.

Thank you for your helpful comments.

**Reviewer #3:** The authors have diligently addressed all comments, and I now find the manuscript suitable for publication in PLOS Medicine. The only minor revision I identified is related to the "What Did the Researchers Do and Find?" section. Specifically, there is duplication of the phrase "limited by" that should be removed. The sentence should read as follows: "Our study is limited by the lack of a cost-effectiveness analysis, data on cotrimoxazole uptake, and comprehensive antimicrobial resistance surveillance data in sub-Saharan Africa."

Thank you for spotting this. We have revised the text [Lines 123-124] to read: “Our study is limited by lack of cost-effectiveness analysis, lack of data on cotrimoxazole uptake, and limited antimicrobial resistance surveillance data in sub-Saharan Africa.”
