# Supplementary material for: Estimating the impact of alternative programmatic cotrimoxazole strategies on mortality among children born to mothers with HIV: A modelling study
Source: PLoS Med. 2024 Feb 20;21(2):e1004334. doi: 10.1371/journal.pmed.1004334 (PMC10914273; doi:10.1371/journal.pmed.1004334)
Supplement: Attachment — Submitted filename: CTX Modelling PLoS Medicine REVISION3 RESPONSE.docx [file pmed.1004334.s006.docx]

Dear Dr Gaynor-Brook and Editorial Office,

We thank you and the reviewers for the thoughtful suggestions and comments.

Please find enclosed the third revision of our manuscript entitled: “Estimating the Impact of Alternative Programmatic Cotrimoxazole Strategies on Mortality Among Children Born to Mothers with HIV: A Modelling Study.” We have further revised the manuscript and we describe the revisions to each of the comments below.

We hope that the revised manuscript is suitable for publication. We look forward to hearing from you.

Sincerely,

Dr Shrey Mathur MD

Comments from the Academic Editor:

In some places in the discussion, the authors could change the order of the relevant sentence to start with insisting that HIV diagnosis and immediate treatment of infants found to be infected is priority, and that provision of cotrimoxazole should not be seen as a 'way out' and reducing the costs of the HIV prevention and care programmes, before stating that co-trimoxazole should be provided to HEU.

Thank you for this comment. We have revised two major sections of the Discussion. In lines 419-421, the first sentence now highlights the importance of strengthening the test-to-treat cascade, and then the paragraph goes on to discuss the safety net that cotrimoxazole provides while that cascade is being strengthened. In the final paragraph of the Discussion (lines 448-451), we have re-ordered it so that we again make the point about the ‘non cotrimoxazole’ elements of PMTCT first, followed by the safety net that cotrimoxazole provides.

RE: availability of trials in breastfed populations - the authors should amend their sentence re no trials being available to note that only limited data is available re use of CTX in HEU in breastfeeding populations, and reference the Mpepu trial, where 20% of infants were breastfed. In many high HIV prevalence settings breastfeeding does happen but often not for that long - for a variety of reasons - which likely underlies the finding in Arikiwa that breastfeeding was not clearly associated with mortality in HEU infants.

Thank you for this suggestion. Please see response to Reviewer #1 below.

There is no need to compare mortality between Mpepu and CHAPS trials.

Thank you.

Requests from the editors:

To help us extend the reach of your research, please provide any Twitter handle(s) that would be appropriate to tag, including your own, your coauthors’, your institution, funder, etc.

Thank you we have added these in the relevant submission field to the Editorial Manager.

Author Summary:

Line 111 - please revise to ‘Increased predicted morality…’ and ‘although it was predicted that cotrimoxazole would have fewer benefits’ or similar

Thank you. We have revised this to read as “Increased predicted mortality persisted in sensitivity analyses considering conservative model estimates, although cotrimoxazole had fewer predicted benefits when vertical transmission rates were lowered, testing coverage improved or uptake of cotrimoxazole was reduced.” [Author Summary, Lines 111-114]

Methods:

Please state early in the Methods section that your study did not have a prospective protocol.

Thank you. We have added to the first paragraph of the Methods section [Lines 182-183]: “The study did not have a prospective protocol.”

Please add the following statement, or similar, to the Methods: "This study is reported as per the Transparent reporting of a multivariable prediction model for individual prognosis or diagnosis (TRIPOD) guideline (S1 Checklist)."

Thank you. We have added to this statement to Methods, Lines 182-184.

Results:

Lines 285, 295, 304, 309 - please revise to ‘predicted excess mortality’ (or similar)

Thank you. We have revised each instance to “predicted excess mortality.” [Lines 288, 298, 307, 312]

Line 320 - please specify which supplementary files are relevant to each setting.

We have amended to include these [Lines 324-325]: “Figures for sensitivity analysis in Cote d’Ivoire (Fig S3.4), Zimbabwe (Fig S3.2), and Uganda (Fig S3.7) are available in the Supplement.”

Figures:

Please ensure that all abbreviations are defined in the figure legend of each figure, including those in the supplementary information.

Thank you. We have added missing abbreviations.

Comments from the reviewers:

**Reviewer #1:** Mathur and colleagues have made further changes to their manuscript entitled "The Impact of Alternative Programmatic Cotrimoxazole Strategies on Mortality Among Children Born to Mothers with HIV: A Modelling Study" in an effort to be responsive to reviewer suggestions. Two key points which were not adequately addressed in this latest revision should be addressed prior to publication.

First, it is again requested that in the limitations paragraph of the discussion section it be pointed out that investment in ensuring that all children HIV-exposed uninfected receive cotrimoxazole until risk of HIV infection has been eliminated may not represent the optimal health investment compared to investing in timely testing and treatment, an alternative known to substantially reduce morbidity and mortality, preserve immune function, and limit seeding of the HIV viral reservoir, the latter two of which cotrimoxazole cannot affect. While the response from the authors to this previous request characterize prescribing of cotrimoxazole as a "safety net" when gaps in the test-to-treat cascade exist, the "safety net" is the health care system. The modeling exercise does not sufficiently account for lack of engagement in the health care "safety net". It is again requested that the wording be clear that funding and programming of cotrimoxazole may not represent the best investment compared to investment in the test-to-treat cascade. It is recommended that this appear in the limitations paragraph of the discussion section.

Thank you for this comment. We have made changes to address this in the Discussion (Lines 419-421 and 448-451) as described above in the response to the comments from the Academic Editor.

Secondly, although the authors do reference the Botswana-based Mpepu study and the South African study, lines 379 through 383 continue to state:

"There are no randomized controlled trial data for cotrimoxazole among breastfed infants, but it is unlikely that mortality benefits are substantially lower than in the original CHAP trial, given the more rapid disease progression at this age, lack of prognostic markers, and higher risk of Pneumocystis jirovecii."

As previously pointed out, the Mpepu study does represent a randomized controlled trial where 20% of the infants in the study were breastfeeding and these infants were randomized to cotrimoxazole or placebo. Please change the statement in the discussion to reflect this and consider comparing Mpepu mortality data to that of the original CHAP trial. If the fact that mortality is only reported through 18 months is the reason Mpepu mortality data was not used, then the text should reflect that rather than indicating that no randomized clinical trial has occurred.

Thank you for this comment. In this section, we are specifically discussing the impact of cotrimoxazole on infants with HIV, and not infants who are HEU. Our point is that CHAP (which randomized cotrimoxazole among children with HIV) included very few infants, and so did not represent the contemporary cohort of breastfed infants with undiagnosed HIV infection – we therefore conducted sensitivity analyses to modify the effect size of cotrimoxazole in case the effect is substantially different. The Reviewer’s comment about 20% breastfeeding in Mpepu is acknowledged, but these infants were all HEU, so it would not address our point about the effect of CTX on mortality in breastfed infants *with HIV.* As the Academic Editor requested, we have not compared mortality between CHAP and Mpepu populations, as it will cause confusion to readers.

We have revised Discussion [Lines 383-387]: “There are limited randomised controlled trial data for cotrimoxazole among breastfed infants with HIV, but it is unlikely that mortality benefits are substantially lower than in the original CHAP trial, given the more rapid disease progression at this age, lack of prognostic markers, and higher risk of *Pneumocystis jirovecii* pneumonia.”

**Reviewer #3:** To enhance the clarity and precision of the manuscript, I propose a revision to lines 410 and 411 in the Discussion section as detailed below:

Lastly, this study did not consider the economic implications of the proposed strategies. Future research should investigate the potential cost savings and the financial impacts of implementing each strategy on national budgets.

Thank you for your helpful suggestion. We have revised to Discussion, Lines 415-417: “Lastly, this study did not consider the economic implications of the proposed strategies. Future research should investigate the potential cost savings and the financial impacts of implementing each strategy on national budgets.”
